# Exploiting the Anticancer, Antimicrobial and Antiviral Potential of Naphthoquinone Derivatives: Recent Advances and Future Prospects

**DOI:** 10.3390/ph18030350

**Published:** 2025-02-28

**Authors:** Shouyan Xiang, Yubei Li, Shah Nawaz Khan, Weixin Zhang, Gaoyang Yuan, Jiahua Cui

**Affiliations:** 1 School of Pharmacy, Gannan Medical University, Ganzhou 341000, China; tc1423177159@sjtu.edu.cn (S.X.); ygy17630814755@163.com (G.Y.); 2 School of Chemistry and Chemical Engineering, Shanghai Jiao Tong University, Shanghai 200240, China; 3 Department of Pharmacy, University of Malakand, Chakdara 18800, Pakistan; 4 School of Agriculture and Biology, Shanghai Jiao Tong University, Shanghai 200240, China

**Keywords:** 1,4-naphthoquinones, anticancer, antimicrobial, antiviral, structure–activity relationships, natural products

## Abstract

Cancer remains a primary cause of mortality, with over 18.1 million new cases and 9.6 million deaths globally in 2018. Chemotherapy, which utilizes a spectrum of cytotoxic drugs targeting the rapidly dividing cancer cells, is a predominant treatment modality. However, the tendency of chemotherapeutics to induce drug resistance and exhibit non-specific cytotoxicity necessitates the development of new anticancer agents with heightened efficacy and minimized toxicity. In recent years, the discovery of safe and effective antibacterial/antiviral agents has also been a hot spot in medicinal chemistry. This paper comprehensively reviews the synthesis, anticancer/antibacterial/antiviral activity, and structure–activity relationships of natural 1,4-naphthoquinones and their derivatives. It highlights their potential as efficient and low-toxicity antitumor and anti-infectious drug candidates.

## 1. Introduction

Cancer is a leading cause of death worldwide, posing a severe threat to human health. Agency for Research on Cancer data from 2022 indicate a staggering 20 million new cases, leading to approximately 9.7 million fatalities globally. China, the most populous nation in the world, occupied almost half of all the cases (49.2%) and the majority of fatalities (56.1%) in the world, implying the severe cancer mortality burden in Asia [1]. Lung, breast, and colorectal cancers are the most prevalent and account for a third of incidence and mortality rates in the world. The primary treatment strategy in clinical settings includes radiotherapy, surgery, and chemotherapy, each playing a vital role in combating these formidable diseases [2].

Malignant tumors present a significant threat to human health, with increasing cancer incidence underscoring the urgency of effective cancer prevention and treatment. In the United States, approximately 1,958,310 new cancer cases are projected for 2023. Looking ahead, the total number of cancer cases in the U.S. is expected to reach 2.3 million by 2025, driven by an aging population. Despite chemotherapy being the primary treatment modality, its extensive cytotoxic effects, poor drug selectivity, and tendency to foster drug resistance pose significant challenges. Consequently, developing highly effective, less toxic antitumor drugs has become a focal point in contemporary antitumor drug research. Addressing these issues is crucial for improving patient outcomes and reducing the global cancer burden.

Chemotherapy is rooted in the principles of cancer biology and molecular genetics. It is considered a cornerstone of cancer treatment and employs a wide range of drugs known for their cytotoxic effects. These drugs primarily, though not exclusively, target the rapidly dividing cancer cells [3]. However, its effectiveness is often overshadowed by significant limitations. Many anticancer drugs lack specificity, leading to collateral damage to healthy cells and causing severe toxic side effects, for example, skin and hair changes, nausea, blood-clotting issues, fatigue, immune suppression, and potential damage to vital organs, with some effects causing long-term harm [4].

A critical challenge in chemotherapy is drug resistance, which accounts for approximately 90% of treatment failures due to cancer’s ability to evade and metastasize [5]. This has steered the scientific community towards developing targeted anticancer therapies that promise higher efficacy and lower toxicity.

The issue of treatment resistance extends beyond cancer. Infectious diseases, notably caused by bacteria and viruses, also pose a global health threat. The COVID-19 pandemic, triggered by the SARS-CoV-2 virus, illustrates this issue significantly, which caused over 591 million cases and 6.43 million deaths by August 2022. Annually, bacterial infections lead to approximately 700,000 deaths, with projections suggesting a rise to 10 million by 2050, surpassing cancer fatalities [6]. The growing challenge of multidrug resistance infections has been recognized by the World Health Organization as one of the top ten global health threats, emphasizing the urgent need for novel therapeutic strategies [7].

The exploration of lead compounds from natural products, followed by their structural modification, forms the cornerstone of drug research and development. Quinone compounds, a notable class of natural organic substances, have substantial medicinal value. Several drugs employed in solid tumor treatments, such as erythromycin, adriamycin, mitomycin, and mitoxantrone, incorporate quinone structures [8,9,10]. Moreover, naphthoquinones, prevalent in plants, fungi, and some animals, exhibit a broad spectrum of pharmacological properties [11].

Among various naphthoquinones, natural 1,4-naphthoquinones, which are naphthol analogs with a conjugated diketone group on the naphthalene structure, are particularly significant. The most crucial subset within this family is 1,4-naphthoquinone, widely utilized as an active pharmaceutical ingredient in the industry. These derivatives display diverse biological activities, including antifungal, antibacterial, antiplatelet, antiallergic, anti-inflammatory, antithrombotic, antiviral, and antitumor activities related to lipoxygenase inhibition, free radical scavenging, and antiradical properties [12,13,14,15].

This article first reviews the synthesis pathways of 1,4-naphthoquinone derivatives, followed by biological activities associated with these compounds, focusing on anticancer, antimicrobial, and antiviral properties. Finally, we discuss these compounds’ structure–activity relationships to understand their mechanisms of action and guide future synthetic efforts.

## 2. Synthesis of 1,4-Naphthoquinones and Its Derivatives

**Figure 1:**
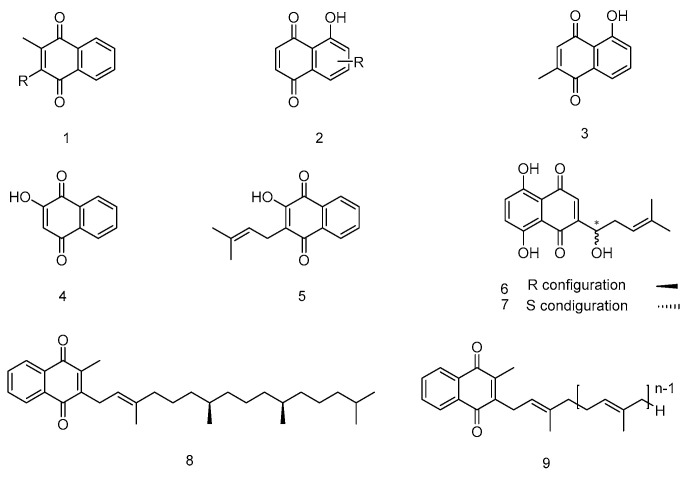
1,4-naphthoquinone analogs. * indicates the chiral carbon in the chemical structure of compounds **6** and **7**, which were in *R* and *S* configuration respectively.

Notable natural 1,4-naphthoquinone products include vitamin K (**1**, Figure 1), juglone (**2**), plumbagin (**3**), lawsone (**4**), lapachol (**5**), shikonin (**6**), and alkannin (**7**) [16,17]. Vitamin K analogs (**1**), characterized by a methyl group on the quinone ring (1,4-naphthoquinone), encompass several natural products (**8**, **9**). Juglone and plumbagin share homologous structures, and similarly, the structures of lawsone (**4**) and lapachol (**5**) are closely related and can be discussed in conjunction. The naturally occurring naphthazarin compounds, such as shikonin and alkannin, are a pair of enantiomers with significant relevance.

### 2.1. Synthesis of Vitamin K and Its Analogs

**Scheme 1:**
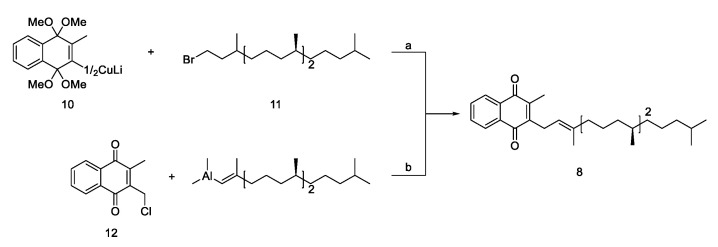
The synthetic routes of vitamin K1 (**8**). Conditions: (a) Phytyl bromide, −60 °C, 30 min, H_3_O^+^; (b) 0.5% (NiCl_2_, PPh_3_, *n*-BuLi), THF, −78 °C, 15 min.

Vitamin K is an essential class of disubstituted 1,4-naphthoquinone derivatives, plays a crucial role in blood coagulation, and is essential for synthesizing coagulation factors in the liver, which can also facilitate the growth of cells [18,19]. There are two naturally occurring forms: vitamin K1 (Phylloquinone, **8**, Scheme 1), an oil-soluble vitamin derived from alfalfa, and vitamin K2 (Menatetrenone, **9**, Figure 1), a crystalline form obtained from fermented fish. Vitamin K1 is abundant in plants and is known as phylloquinone due to its phytane side chain [20,21,22]. Vitamin K2, the biologically active form of vitamin K, is subdivided into various subtypes like MK-4 and MK-7, differentiated by the number of isoprene units at the 3-position, the two most studied subtypes [23].

Vitamin K1 (**8**) isolation from alfalfa leaves opened the doors for different synthetic approaches involving Friedel–Crafts acylation using phytobromine or chlorophyll alcohol and 2-methyl-1,4-naphthoquinone [24,25]. While presenting a straightforward strategy, these methods result in low yields [26,27,28]. Subsequent optimizations by Chenard [29] and Lipshutz [30] significantly improved yields. Chenard’s method involved coupling protected naphthoquinone cuprate (**10**) with phytyl bromine (**11**), while Lipshutz’s approach used chloromethylated 2-methyl-1,4-naphthoquinone (**12**) coupled under nickel chloride and triphenylphosphine catalytic conditions.

**Scheme 2:**
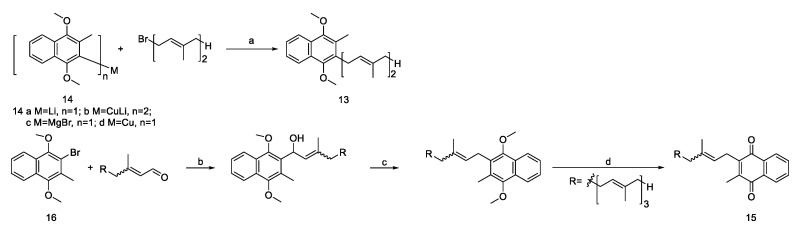
The synthetic route of vitamin MK-4. Conditions: (a) AgO, H^+^; (b) *n*-BuLi, THF; (c) Li**/T**HF; (d) Ce (IV).

For the synthesis of vitamin MK-2 (**13**, Scheme 2), Snyder et al. [31] reported a method using 2-metal-3-methyl-1,4-dimethoxynaphthalene (**14**) and brominated alkanes, proving most effective for high-yield MK-2 synthesis up to 80% and 99% retention of E-configuration. Garcias [32] described a synthesis route for MK-4 (**15**), involving 2-bromo-3-methyl-1,4-dimethoxynaphthalene (**16**) and allyl aldehyde coupling using n-butyllithium to deliver corresponding alcohol, followed by Birch hydrogenolysis (BIHY) to retain the conformation of a desired intermediate and, finally, ceric ammonium nitrate (IV) oxidation to obtain vitamin MK-4.

**Scheme 3:**
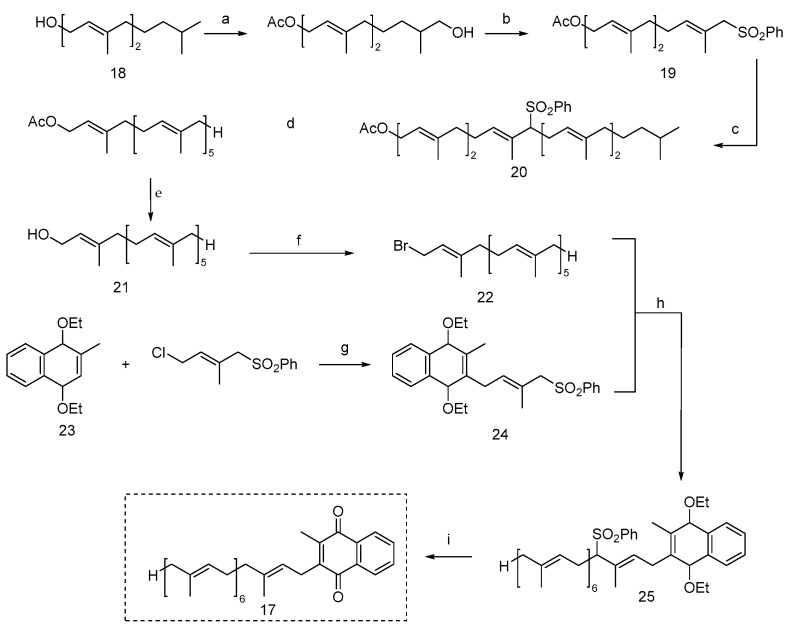
The synthetic route of vitamin MK-7. Conditions: (a) Ac_2_O, Py, SeO_2_, *^t^*BuO_2_H, salicylic acid; (b) PBr_3_, PhSO_2_Na; (c) *^t^*BuOK, Farnesyl bromide; (d) LiEt_3_BH, Pd(dppe)Cl_2_; (e) OH; (f) PBr_3_, CH_2_Cl_2_; (g) SnCl_4_, CH_2_Cl_2_, 0 °C; (h) NaHMDS, THF, −20 °C; (i) CAN, CH_3_CN/CH_2_Cl_2_.

Vitamin MK-7 (**17**, Scheme 3), a subtype of vitamin K2, has distinguished high biological activity and better bioavailability than other vitamin K forms. It features minimal intestinal absorption and is retained in the serum for up to three days. Such efficiency allows small daily doses to suffice in supporting vitamin K-dependent enzymes and proteins. MK-7 also plays a role in calcium metabolism, contributing to bone formation and influencing the health of arterial blood vessel walls, unlike vitamin K1.

The synthesis of vitamin MK-7, based on its molecular structure, can be achieved from menadione or its protected derivatives. In a notable advancement, Krajewski et al. [33] developed a multistep synthesis process, which has been the subject of a U.S. patent application. The synthesis begins with farnesol (**18**, Scheme 3), which undergoes acetylation, followed by hydroxyl group oxidation using selenium dioxide. The resulting product is then esterified using phosphorus tribromide, leading to intermediate **19**. Then, the substitution reaction with brominated farnesol formed intermediate **20**, and the substitution of benzenesulfonyl and acetyl groups yielded intermediate **21**. The next step involves bromination to produce key intermediate **22**. Concurrently, diethylmethanaphthoquinone (**23**) undergoes a substitution reaction to form intermediate **24**. The intermediates **24** then undergo a nucleophilic substitution to deliver intermediate **24**. Finally, dephenylsulfonylation and oxidation culminate in the production of vitamin MK-7.

### 2.2. Synthesis of Juglone (5-Hydroxy-1,4-Naphthoquinone)

Lawsone and Juglone differ in the position of their hydroxyl groups, impacting their synthesis and biological activities. This structural difference influences the electron density distribution in each molecule, contributing to distinct bioactivity profiles.

**Scheme 4:**
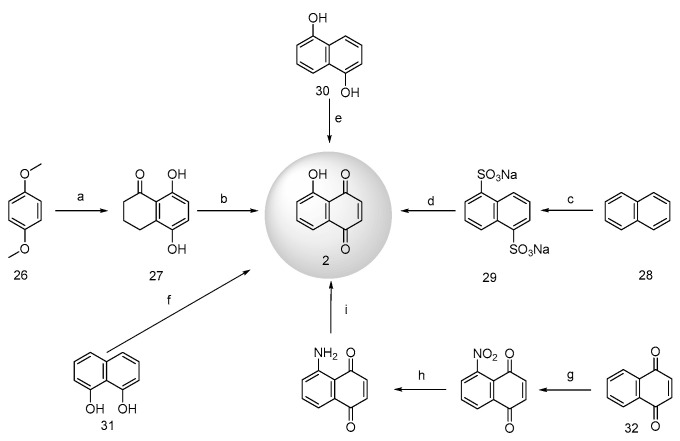
The synthetic routes of juglone. Conditions: (a) Succinic anhydride, PhNO_2_, AlCl_3_, Et_3_SiH, CF_3_COOH, HBr, reflux; (b) DDQ, Benzene, reflux; (c) H_2_SO_4_, SO_3_, 55 °C; (d) NaOH, 330 °C, H_2_SO_4_, Na_2_Cr_2_O_7_, H_2_O, 15 °C; (e) Ac_2_O, H_2_O_2_, MeOH, 50 °C; (f) HIO_4_, microwave, 50 °C; (g) HNO_3_, H_2_SO_4_; (h) Na_2_S_2_O_4_, then H_2_O_2_; (i) NaNO_2_, then Cu^2+^.

Juglone (**2**, Scheme 4), scientifically denoted as 5-hydroxy-1,4-naphthoquinone, is an intrinsic dye compound naturally found in Lawsonia inermis leaves and widely distributed across the roots, husks, fruits, and bark of the walnut family [34,35]. However, due to its low content and extraction efficiency in *P. nutans*, chemical synthesis is the predominant source of juglone [36].

The early synthesis of juglone was achieved through a Friedel–Crafts acylation and cyclization reaction utilizing 1,4-dimethoxybenzene (**26**) as the foundational substrate, forming an intermediate (**27**). Khalafy et al. [37] pioneered juglone synthesis, yielding the intermediate 5,8-dihydroxy-1-tetrahydronaphthalenone (**27**), which was subsequently oxidized using DDQ to yield juglone. Notably, this method offers relatively high yields; however, its dependence on toxic solvents, such as benzene, renders it useless for industrial-scale production.

Subsequent methodologies harnessed juglone’s quinone structure [38], focusing on the oxidation of naphthalene and naphthol as critical intermediates. Mamchur et al. [39] employed naphthalene (**28**) as the primary substrate, initially using sulfonation to form a salt, followed by alkalinization to obtain a phenol sodium salt, and ultimately subjecting it to oxidation with dichromate to yield juglone. However, it is a straightforward synthetic strategy. The main disadvantages are low yields and the use of heavy metals like chromium, which presents environmental contamination concerns.

Mingxiao Zhang et al. [40] introduced an innovative one-pot synthesis method for juglone. This approach synthesized the peracetic acid from acetic anhydride and hydrogen peroxide, eliminating the need for sulfuric acid. Subsequently, the authors incrementally added a methanolic solution of 1,5-dihydroxynaphthalene (**30**) and stirred at 50 °C for 4 h to yield juglone. The main advantages of this method are ease of operation, high yields, cost-effectiveness, readily accessible raw materials, and eliminating the necessity for toxic solvents. Iodylbenzene or iodosylbenzene employed as oxidants to obtain juglone was also a sophisticated method reported by R. Barret and M. Daudon [41]. Michael Oelgemoller et al. [42] employed a photochemistry protocol, oxidizing 1,5-dihydroxyl naphthalene to harness juglone in a special reactor. This method was environmentally friendly, with only oxygen gas and sunlight required, and produced little waste.

Hashemi et al. [43] embraced an environmentally conscientious synthetic technique, solid-phase microwave oxidation, for juglone synthesis. The method entailed mixing 1,8-dihydroxynaphthalene (**31**), periodate, and kaolinite clay in a mortar, followed by microwave irradiation for 50 s, resulting in a commendable 91% yield of juglone. This approach has a brief reaction duration, high yield, and negligible environmental impact. Hong Li et al. [44] reported a method employing 1,4-naphthoquinone **(32**) as the starting material, nitrified the α-H of the naphthoquinone. Reduction in the nitro group, diazotization, and hydrolysis procedure afforded the final product juglone subsequently.

### 2.3. Synthesis of Lawsone (2-Hydroxy-1,4-Naphthoquinone)

Lawsone, also known as 2-hydroxy-1,4-naphthoquinone, is a reddish-orange dye naturally occurring in the leaves of *Lawsonia inermis* and the flowers of water hyacinths. Its historical use as a hair and skin dye dates back over 5000 years [45]. Lawsone can form a Michael addition reaction with the protein keratin in the skin and hair, resulting in long-lasting coloration until natural shedding occurs. Furthermore, it exhibits strong UV-absorbing properties, making aqueous extracts effective in preventing skin tanning. Due to its susceptibility to biodegradation during extraction, contemporary production primarily relies on chemical synthesis [33,46,47].

**Scheme 5:**
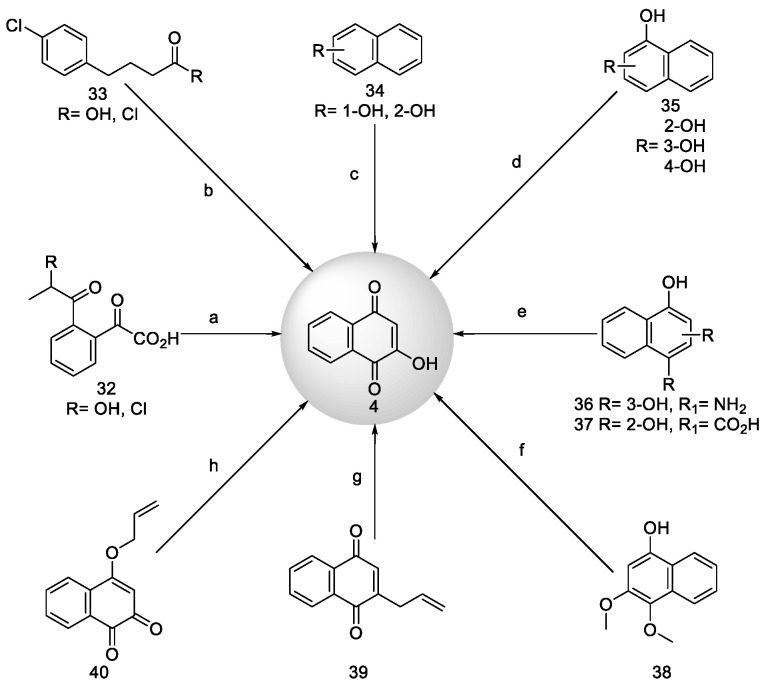
The synthetic routes of lawsone. Conditions: (a) *p*-Nitrotoluene, CuSO_4_ or NaOH, air; (b) HCl, NaOH, AlCl_3_, O_2_, PdCl_2_, MeOH, sod. tetrahydroborate, CS_2_, CH_2_Cl_2_; (c) KO_2_, 18-crown-6, toluene; (d) crown-ether/pyridine or KO_2_/THF, −10 °C; (e) [O], H_2_O, HCl or aq. KOH, air; (f) Prussian red, H_2_O, NaOH; (g) H_2_O_2_, KOH; (h) basic hydrolysis.

Fieser et al. [48] accomplished the synthesis of lawsone (**4**, Scheme 5) through the closure of the phenyl derivative’s ring (**32**) via a cyclization reaction. This reaction can occur under alkaline conditions in atmospheric air or catalyzed by *p*-nitrotoluene and copper sulfate. Inoue et al. [49] employed a multistep process to convert 4-chlorobutyryl compounds (**33**) into lawsone. Initially, naphthol (**34**, **35**) was oxidized to 2-hydroxy-1,4-naphthoquinone using potassium superoxide (KO_2_) in a toluene solution of 18-crown-6 [50] or a tetrahydrofuran solution at −10 °C [51], delivering a 34% yield. Subsequently, 4-amino-1,3-naphthalenediphenol (**36**) underwent oxidation to deliver lawsone under HCl or NaOH conditions at 45 °C for an hour [52]. 3,4-dihydroxy-1-naphthoic acid (**37**) was hydrolyzed under alkaline KOH conditions in the presence of air to yield the target product [53]. Further oxidation of 3,4-dimethoxy-1-naphthol (**38**) was carried out in aqueous NaOH and potassium ferricyanide to afford lawsone (65% yield) and a dimer product [54]. Subsequently, 2-allyl-1,4-naphthoquinone (**39**) underwent oxidation using KOH and H_2_O_2_, yielding a lawsone. Finally, the hydrolysis of 4-allyloxy-1,2-naphthalenedione (**40**) under alkaline conditions resulted in the formation of lawsone and allyl alcohol.

### 2.4. Synthesis of Shikonin and Alkannin

**Scheme 6:**
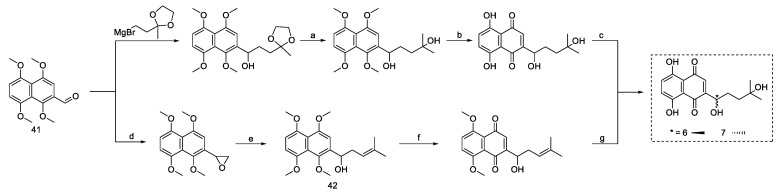
The synthetic route of racemic shikonin. Conditions: (a) TsOH, CH_3_COCH_3_, MgMeI, THF; (b) CAN, CH_3_CN/CH_2_Cl_2_, AgO, AgNO_3_; (c) AcOH, Py, SOCl_2_, NaOH, CH_3_COOH; (d) Me_2_SCH_2_; (e) isopropyl thin lithium; (f) CAN, CH_3_CN/CH_2_Cl_2_; (g) AgO, AgNO_3_.

Red-root gromwell, belonging to the Comfrey family, encompasses two distinct species: *Lithospermum erythrorhizon* Sieb. & Zucc. and *Arnebia euchroma* (Royle) Johnst. It is predominantly found in regions spanning China, Korea, and Japan [55]. Within this botanical genus, shikonin (**6**, Scheme 6) and alkannin (**7**) emerge as a pair of enantiomeric isomers distinguished by their naphthazarin structure. Shikonin serves as the primary active constituent of Lithospermum erythrorhizon, whereas alkannin takes precedence in Arnebia euchroma. While conventional methods for obtaining shikonin have traditionally involved plant tissue extraction or cellular tissue cultivation, sustained research efforts have now made it feasible to achieve high enantiomeric excess (ee) values of shikonin, alkannin, and their derivatives through chemical synthesis [56,57].

Most synthetic approaches to shikonin rely on 1,4,5,8-tetramethoxynaphthaldehyde as a key starting material. Terada et al. [58] made a pioneering contribution by synthesizing racemic shikonin, commencing with 1,4,5,8-tetramethoxynaphthaldehyde (**41**) as the initial substrate. The method involves a nucleophilic addition reaction of a glycol-protected Grignard reagent to the aldehyde, followed by subsequent hydrolysis, ketone oxidation, and, ultimately, demethylation in the presence of AgO/AgNO_3_ to yield racemic shikonin. Subsequently, Moiseenkov et al. [59] employed a similar strategy using 1,4,5,8-tetramethoxynaphthaldehyde (**41**) as the starting material. This method encompassed epoxidation, followed by ring opening through a reaction with organic lithium reagents, forming 1,4,5,8-tetramethoxy shikonin (**42**). The final step involved racemic shikonin synthesis through oxidation with ceric ammonium nitrate and demethylation with AgO/AgNO_3_.

**Scheme 7:**
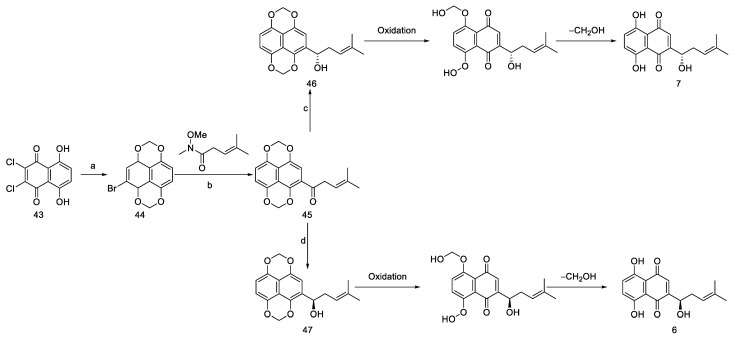
The synthetic routes of shikonin and alkannin. Conditions: (a) SnCl_2_, HCl, BrCH_2_Cl, K_2_CO_3_, DMF, NBS, CHCl_3_; (b) *t*-BuLi, THF; (c) (+)-DIP-Cl, THF; (d) (−)-DIP-Cl, THF.

Nicolaou et al. [60] have meticulously documented a sophisticated total synthesis strategy aimed at elucidating the absolute conformation of shikonin (**6**, Scheme 7) and alkannin (**7**). The method commences with the strategic utilization of commercially available 2,3-dichloronaphthazarin (**43**) as the primary starting material. This strategic approach involves the initial protection of the hydroxyl group, concomitant with the simultaneous bromination of the naphthazarin ring, culminating in the formation of intermediate **44**. Subsequently, introducing the shikonin side chain at the brominated position yields the aryl ketone intermediate **45**. This intermediate is of paramount significance, as it is an exceptionally well-suited substrate for numerous asymmetric reducing agents, rendering it the optimal choice for synthesizing both enantiomers. The reduction step, meticulously orchestrated through utilizing DIP-Cl, a chiral reducing agent, leads to the formation of intermediates **46** (S-configuration) and **47** (R-configuration), respectively. These intermediates are further subjected to oxidation and the subsequent removal of formaldehyde, ultimately revealing the absolute configurations of alkannin and shikonin.

This groundbreaking methodology stands out on several fronts. Firstly, it harnesses naphthazarin’s economic and abundant resources as the primary starting material, contributing to its overall feasibility and accessibility. Secondly, it ingeniously generates an intermediate tailored to synthesize alkannin and shikonin with exceptionally high enantiomeric excess (ee) values with a convenient method to operate in the industry, attesting to its precision and efficiency. Lastly, the protective strategy demonstrates remarkable ingenuity, facilitating a straightforward, one-step deprotection process under mild conditions, underscoring its practicality and elegance.

In parallel, Rubing Wang et al. [61] have undertaken a study of utmost relevance, successfully yielding R and S configuration intermediates through the strategic introduction of S-1-phenylethylamine at the chiral carbon. Subsequent multistep hydrolysis and reduction reactions culminated in the acquisition of shikonin and alkannin with extraordinarily high ee values, underscoring their exemplary craftsmanship and commitment to scientific rigor.

This section introduced several typical naphthoquinone synthesis routes, most of which are multistep protocols. However, the common place among them was the construction of the naphthoquinone moiety. Apparently, the most convenient strategy to synthesize naphthoquinone was the oxidation of the naphthalene, which provides insight into the oxidation reagent. Standard oxidation reagents, including cerium ammonium nitrate (CAN), Ag_2_O, periodic acid, oxygen, KO_2_ and H_2_O_2_, could oxidize the hydroxyl naphthalene or methoxy naphthalene to naphthoquinone. Currently, the most widely used reagent for oxidation is CAN, which is relatively optimal since the condition is mild and the cost is relatively low.

**Scheme 8:**
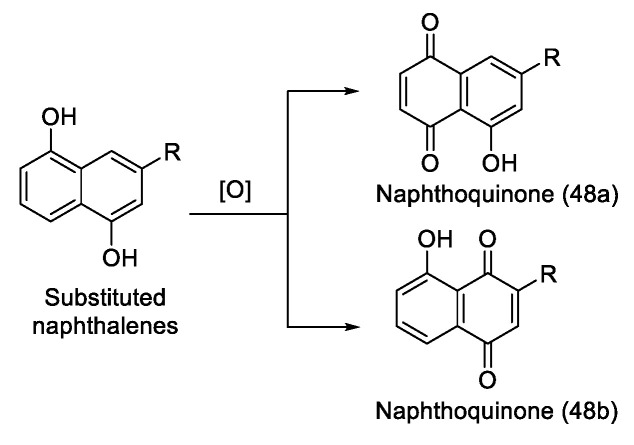
Oxidation strategy of naphthalene.

However, the presence of substituents on the naphthalene ring can lead to regioselectivity challenges during oxidation. As shown in Scheme 8, oxidation may occur at different positions, resulting in the formation of regioisomeric naphthoquinones (for example, **48a** and **48b**). These regioisomers differ in the position of the quinone groups rather than being enantiomers, as they do not exhibit chirality. Developing a novel oxidation strategy that improves regioselectivity could significantly enhance the efficiency of naphthoquinone synthesis.

## 3. Biological Activity of 1,4-Naphthoquinone Derivatives

1,4-Naphthoquinone derivatives induce cytotoxicity through mechanisms like redox cycling, arylation, DNA strand breakage, free radical generation, and quinone methylation. These compounds, featuring quinone structures, inhibit specific proteins, resulting in antibacterial, antiviral, and antitumor effects. Agents like adriamycin, mitoxantrone, and compounds like lawsone, β-lapachol, and shikonin primarily inhibit topoisomerase I for antitumor activity [61,62,63].

### 3.1. Anticancer Activity

DNA topoisomerases control DNA’s topological structure by precisely breaking and rejoining DNA strands. They fall into two major classes: topoisomerase I and topoisomerase II. Mammalian topoisomerase II is a crucial target in clinical tumor therapy.

The tumor suppressor gene p53 responds to a myriad of both intracellular and extracellular cues, such as growth factor deprivation, hypoxia, radiation, chemical deficiencies, and nucleotide synthesis aberrations. Upon activation, p53 predominantly instigates apoptosis through transcriptional regulation, eliminating cells with severely damaged DNA. Alternatively, it can initiate growth arrest, facilitating DNA repair and preventing tumor formation [64,65].

The c-Jun N-terminal kinase (JNK), a member of the mitogen-activated protein kinase family, is pivotal in the signal transduction pathway responsible for conveying extracellular signals into intracellular responses. It is crucial in various physiological processes, including cell growth, differentiation, and apoptosis [66]. JNK can also induce apoptosis in response to different stresses, primarily by altering gene expression and initiating the phosphorylation of specific substrates. Furthermore, JNK activation leads to the phosphorylation of p53 and its stabilization, achieved by disrupting its binding to MDM2. Numerous studies have established that JNK activation is essential for stress-induced mitochondrial cytochrome release and apoptosis through the mitochondrial caspase-9 pathway. Moreover, the phosphorylation of JNK enhances the pro-apoptotic activity of multiple factors [67].

Vitamin K2 serves a critical function by facilitating the carboxylation of primary osteocalcin secreted by osteocytes, thereby promoting the deposition of calcium ions from the bloodstream into bone tissue. It is pivotal in the blood coagulation cascade and bone formation [68]. Recent research by Elikan Mishima et al. [69] has unveiled vitamin K’s potential as a ferroptosis inhibitor, suggesting its application in treating organ injuries, therapy-resistant cancers, Parkinson’s disease, and Alzheimer’s disease. However, due to its limited absorption, substantial and frequent doses of the synthetic MK-4 type of vitamin K2 must meet the average body requirements. Additionally, a recent study has highlighted the substantial anticancer effects of vitamin K2, showing its synergistic potential when combined with various anticancer agents [70]. Furthermore, research into various vitamin K analogs with different substituents is ongoing, contributing to developing new anticancer agents.

**Figure 2:**
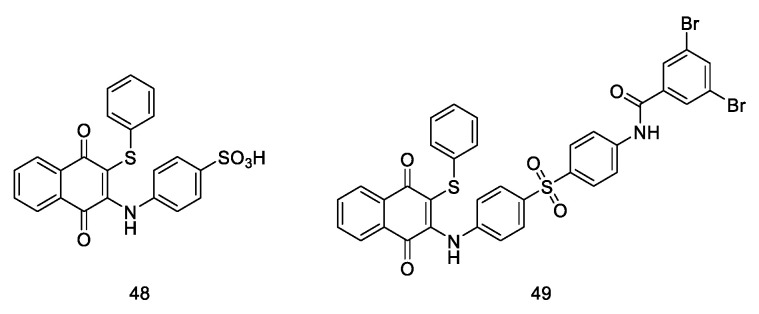
Phenylthiosulfone contained 1,4-naphthoquinone derivatives as anticancer agents.

Yoo et al. [71] introduced phenylaminophenylthiosulfone compounds, incorporating an acyl group at the 3-position and a benzenethiol group at the 2-position of the naphthoquinone core scaffold, resulting in the synthesis of **48**–**49** (Figure 2). These compounds underwent rigorous in vitro cytotoxicity assessments using three distinct cell lines: MCF-7, A549, and HeLa. Furthermore, **49** (IC_50_ = 40 μM) demonstrated robust cytotoxic effects against HeLa, comparable to doxorubicin (IC_50_ = 30 μM). An in-depth structure-activity relationship analysis underscored the critical role of the 3-position substitution for antitumor selectivity, while the 2-position benzenethiol group substitution significantly enhanced cytotoxic activity.

**Figure 3:**
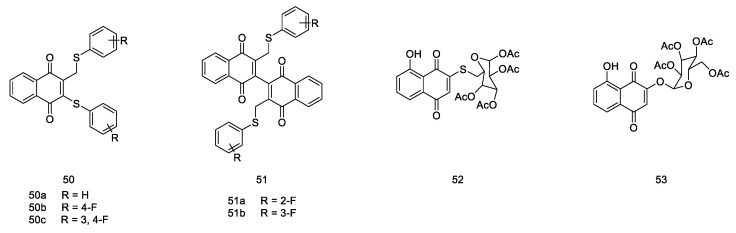
Sulfur-containing 1,4-naphthoquinone derivatives as anticancer agents.

In a pioneering study, Wellington et al. [72] synthesized a novel series of vitamin VK3 analogs, innovatively incorporating sulfur-containing side chains at either the 2- or 3-position of the naphthoquinone moiety. This modification was geared towards enhancing anticancer efficacy. The research team rigorously tested these compounds against a diverse six-cell line panel to evaluate their anticancer activities. The findings were substantial. Compounds **50**–**51** (Figure 3) exhibited a remarkable inhibitory effect on cancer cells, surpassing the effectiveness of traditional vitamin VK3, as indicated by their lower GI_50_ values. Crucially, these compounds demonstrated heightened selectivity for cancer cells over normal human cells (specifically WI-38 cells), a significant advancement in targeted cancer therapy. This selectivity reduces potential collateral damage to healthy cells, a critical consideration in chemotherapy. A significant aspect of Wellington et al.’s research was the exploration of structure-activity relationships within these compounds. Introducing a fluorinated phenyl group at the 1,4-naphthoquinone sulfide moieties significantly boosted their anticancer activity.

Additionally, the study shed light on the potent antitumor properties of the 1,4-naphthoquinone sulfide dimer. In a comparative analysis of compounds **51a** and **51b**, a critical observation was made: the fluorine atom positioned at the 4-position was instrumental in enhancing the anticancer activities of these compounds. This insight underscores fluorination’s impact in elevating anticancer agents’ efficacy.

Wellington et al.’s research represents a significant stride in anticancer drug development. It exemplifies the potential of structural modifications in drug design and sets a precedent for future research in developing more effective and selective anticancer therapies.

Yu-Pu Juang et al. [73] conceived and synthesized a batch of juglone derivatives as the cell surface PDI (protein disulfide isomerase) inhibitor and evaluated their anticancer activity. The cytotoxicity experiments showed that compound **52** (Figure 3) exhibited distinct activity against several cell lines, including MDA-MB-231 (IC_50_ = 6.96 ± 0.47 μM), RPMI8226 (IC_50_ = 0.57 ± 0.01 μM), and EAhy926 (IC_50_ = 7.27 ± 0.08 μM). Additionally, compound 53 also showed remarkable cytotoxic activity against the tested cell lines, especially RPMI8226 (IC_50_ = 0.66 ± 0.08).

**Figure 4:**
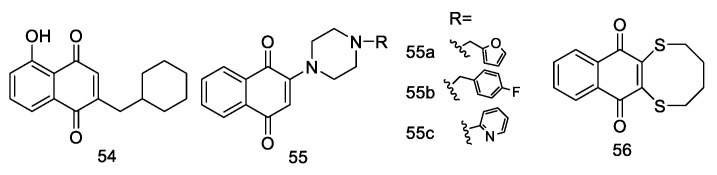
**Nitrogen and Sulfur modified** 1,4-naphthoquinone derivatives as anticancer agents.

Gokmen et al., in their significant research study [74], embarked on the synthesis of a series of N-substituted and S,S-substituted naphthoquinone derivatives (**55**, **56**, Figure 4), aiming to explore their potential as cytotoxic agents against various cancer cell lines (A-549, DU145, HCT-116, and MDA-MB-231 cancer cells), representing a diverse set of neoplastic conditions.

Suresh Awale et al. [75] conceived and synthesized a series of plumbagin derivatives, among which compound 60 showed a distinct anticancer activity against PANC-1 (IC_50_ = 47.2 μM in DMEM; IC_50_ = 0.11 μM in NDM). In vivo, the antitumor evaluation indicated that the plumbagin derivative **54** possessed potent inhibition activity against pancreatic tumor growth. With the treatment of compound **54**, at the dosage of 50 μg/day and 250 μg/day, the weight of the tumor was suppressed (205.8 and 107.6 mg, respectively), compared with the control group’s 465.4 mg.

The results of their study were notable. Compounds **55a** and **56,** as illustrated in Figure 4, demonstrated considerable cytotoxic activity across the examined cell lines. These findings are significant, as they suggest a broad-spectrum efficacy of these compounds against different types of cancer cells. In addition, compound **55b** exhibited specific inhibitory effects against MDA-MB-231 cells. Furthermore, compound **55c** showed a distinct inhibition profile, effectively targeting MDA-MB-231 and DU-145 cells.

Khoshneviszadeh et al. [76] synthesized a novel series of aminonaphthoquinone compounds, distinctively integrating 1,2,3-triazole hybrids at the 2-position of the naphthoquinone ring. The primary aim was to evaluate the cytotoxic activity of these compounds against three human cancer cell lines: MCF-7 (breast cancer), HT-29 (colorectal cancer), and MOLT-4 (acute lymphoblastic leukemia).

**Figure 5:**
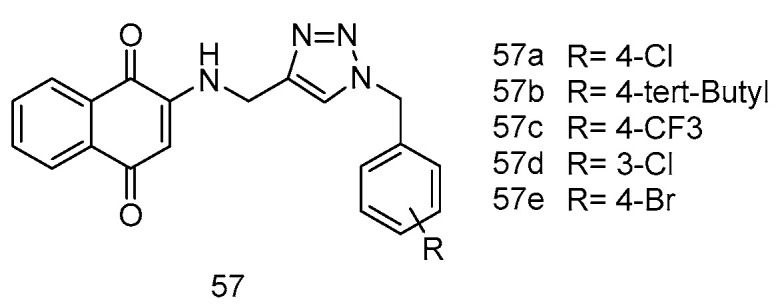
Benzyl clicked 1,4-naphthoquinone derivatives as anticancer agents.

Among the synthesized compounds, **56c** (Figure 5) emerged as the most effective, demonstrating potent anticancer activity across all three cell lines. This efficacy is quantified by its IC_50_ values, 10.4 μM for MCF-7, 6.8 μM for HT-29, and 8.4 μM for MOLT-4. These values suggest a notable capacity of compound **56c** to inhibit cancer cell proliferation at relatively low concentrations.

A critical aspect of the study was SAR studies to enlighten the cytotoxicity effects of different compounds. It was observed that the presence of a halogen atom on the benzene ring notably increased the cytotoxic potency of the 1,2,3-triazole hybrids. This was particularly evident when comparing compound 61c with its counterparts **57a**, **57b**, **57e**, and **57d.** Such findings underscore the impact of minor structural modifications on the biological activity of these compounds.

In general, this work contributes significantly to the field of anticancer drug design, particularly in highlighting the therapeutic potential of aminonaphthoquinone derivatives. The study demonstrates the capability of these compounds to target various cancer cell lines. It offers valuable insights into the role of structural elements, such as halogenation, in enhancing cytotoxic effects. This work paves the way for future explorations into more effective and targeted cancer therapies.

**Figure 6:**
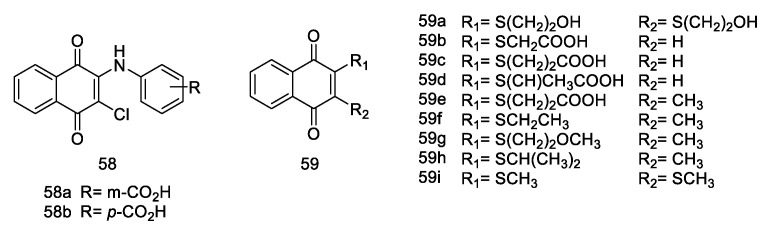
Sulfur-modified 1,4-naphthoquinone derivatives as anticancer agents.

The study by Kirpotina et al. [77] ventures into the realm of cellular signaling, explicitly targeting the cell division cycle 25 (Cdc25) and Mitogen-Activated Protein Kinase Kinase 7 (MKK7), both intimately linked to tumor development. The authors explored this by introducing a phenylamino side chain at the 2-position of the naphthoquinone ring, resulting in two structurally similar compounds, **58** and **59** (Figure 6). Their investigation further expanded to a series of naphthoquinone compounds (**59a**–**i**) with diverse substituent groups, evaluating their anticancer activities against nine tumor cell lines, inhibitory effects on MKK7 and MKK4, as well as Cdc25 A/B, complemented by molecular docking studies.

In their findings, compounds **59a** and **59f**–**i** stood out for their notably low IC_50_ values, indicating potent cytotoxicity. Among these, compound **59c** emerged as a significant inhibitor of MKK7, showcasing an IC_50_ value as low as 0.23 μM. Compounds **59b**, **59c**, **59d**, and **58a** also demonstrated substantial inhibitory potential against both MKK4 and MKK7. In the context of Cdc25A and Cdc25B inhibition, compounds **59a**, **59b**, **59i**, and **58b** were particularly notable. The correlation between the inhibition of Cdc25A/B and the anticancer activity was evident, suggesting a potential pathway for therapeutic targeting. Conversely, the study observed no clear correlation between the anticancer activity and inhibition of MKK7.

This detailed investigation by Kirpotina et al. contributes significantly to our understanding of the structure-activity relationships, specifically in cancer-signaling pathways. Their work underscores the potential of naphthoquinone derivatives in targeted cancer therapy, offering insights into the structural determinants of their biological efficacy. The lack of correlation between MKK7 inhibition and anticancer activity also opens new avenues for understanding the complex mechanisms of tumor suppression and developing more effective cancer treatments.

**Figure 7:**
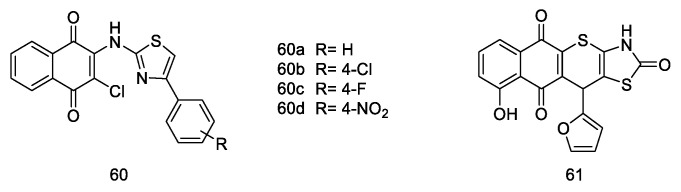
**Thiazole** 1,4-naphthoquinone derivatives as anticancer agents.

Olawode et al. [78] synthesized a series of naphthoquinone compounds, innovatively substituted with a 2-chloro-3-(thiazol-2-yl) amino group. Their research aimed to explore the compounds’ multifaceted biological activities, including anticancer, antimycobacterial, and antimalarial effects. Notably, **60a** (IC_50_ = 1.8 µM), **60b** (IC_50_ = 2.7 µM), **60c** (IC_50_ = 1.5 µM), and **60d** (IC_50_ = 0.004 µM) demonstrated significant cytotoxicity against SH-SY5Y neuroblastoma cells, as evidenced by their low IC_50_ values (Figure 7). Derivative **60d** reveals a significant cytotoxic activity that can contribute to the electronegative substitution at the phenyl group. However, these compounds exhibited minimal inhibition against HeLa cervical adenocarcinoma cells, indicating selective cytotoxicity towards SH-SY5Y cells. This selectivity is critical, as it suggests potential avenues for targeted neuroblastoma therapy, a vital aspect of personalized medicine in oncology.

Iryna Ivasechko et al. [79] received inspiration from thiazole and naphthoquinone compounds. They designed a series of naphthoquinone derivatives to determine the anticancer activity in vitro of condensed hybrid 4-thiazolidinone derivatives and their DNA interaction and general toxicity in vivo. Derivative **61** (Figure 7) with furan out of the condensed 4-thiazolidinone moiety showed relatively high antitumor activity, and the highest IC_50_ value was up to 0.6 μM. Furthermore, they also demonstrated that **59** could increase the percentage of cells in the G2/M phase of the cell cycle and reduce the synthesis of DNA and RNA in tumor cells.

**Figure 8:**
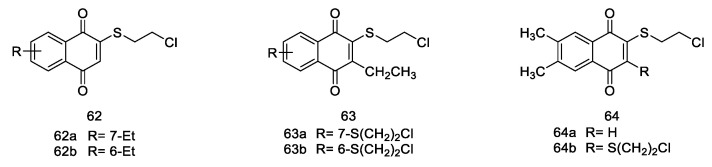
2-chloroethylthio substituted 1,4-naphthoquinone derivatives as anticancer agents.

Dyshlovoy and Pelageev et al. [80] approached naphthoquinone synthesis by introducing a 2-chloroethylthio group onto the naphthoquinone moiety. They tested the resulting compounds against advanced prostate cancer cell lines, uncovering that **62**, **63**, and **64** (Figure 8) exhibited potent inhibitory effects. Derivatives **62** and **64a**, in particular, showed remarkable activity and selectivity, with IC_50_ values ranging from 0.14 μM to 0.77 μM. The study provides valuable insights into the structural determinants of cytotoxicity, indicating that introducing the 2-chloroethylthio group enhances the compounds’ anticancer potential. Moreover, the research demonstrated that **62** and **64b** exert their anticancer effects primarily through the induction of DNA damage and cysteinase-dependent apoptosis, offering a novel therapeutic mechanism against prostate cancer.

**Figure 9:**
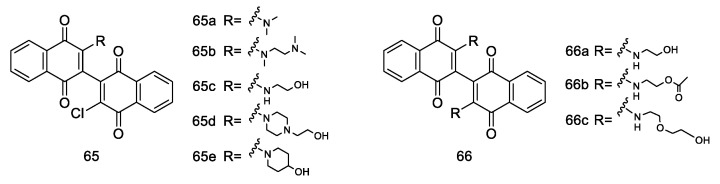
Naphthoquinone dimers as anticancer agents.

Ferraris et al. [81] explored a unique approach by dimerizing amino alcohol-substituted naphthoquinone compounds. Their investigation focused on the compounds’ anticancer activities against Acute Myeloid Leukemia cell lines, including MOLM-14 and MV4-11. The study highlighted **65a-e** presenting a remarkable anticancer activity (Figure 9), along with **65d**, **66a,** and **66b** exhibiting pronounced cytotoxic effects. The findings also revealed that **66a** and **66b** inhibited Indoleamine 2,3-dioxygenases (IDO), suggesting a mechanism that disrupts cancer cell proliferation, a significant advance in understanding the molecular pathways of these compounds.

**Figure 10:**
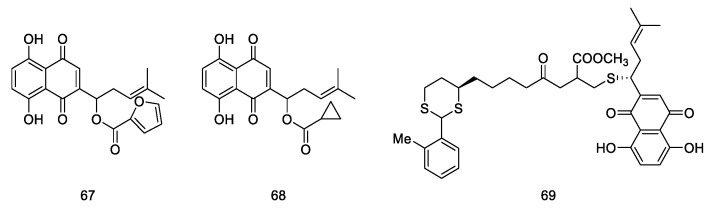
Shikonin derivatives as anticancer agents.

Shikonin is a naturally occurring compound capable of excreting profound antitumor, antitelomerase, and antiangiogenesis effects and is also responsible for DNA topoisomerase I/II inhibition [82,83]. A pair of enantiomers, identified as alkannin and shikonin, were isolated and identified from the roots of two different plants: *Alkanna tinctoria* (L.) *Tausch* in Europe and *Lithospermum erythrorhizon Sieb. et Zucc*. in the Orient, respectively. These compounds demonstrated remarkable antiproliferative properties, making them attractive lead compounds in developing anticancer drugs. Nevertheless, alkannin/shikonin and their synthetic counterparts were not employed in clinical trials as anticancer medications. Their non-specific cytotoxicity in vitro and in vivo posed the biggest challenge. In the literature, it has been demonstrated that reactive oxygen species (ROS) production and bioreductive alkylation are intimately linked to the mechanism of shikonin’s anticancer activity and widespread toxicity. It has been demonstrated that the cytotoxicity caused by ROS and alkylation is non-specific, impacting a wide range of biological macromolecules that include key proteins and nucleic acids in cellular biology, thus not only destroying cancer cells but also impacting healthy cells. Therefore, the structural modification of shikonin derivatives is a highly desirable strategy for medicinal chemists. Yang et al. [84] modified shikonin, a compound known for its antitumor properties, by introducing furoic acid on its hydroxyl group side chain. This modification resulted in a novel derivative **67** (Figure 10) exhibiting enhanced topoisomerase II inhibitory activity and substantial cytotoxicity (ave. IC_50_ value of 7.75 µM) against a range of cancer cells, including drug-resistant variants. This derivative also showed effectiveness in vivo against several human carcinomas. Complementing this, Durchschein et al. [17] developed new shikonin derivatives, discovering that **68** displayed robust inhibitory activity against melanoma cells (WM164 and MUG-Me12) and induced apoptosis in a caspase-dependent manner without causing cell membrane damage or cell cycle arrest, highlighting a distinct mechanism of action.

Hongyan Lin et al. designed and synthesized a series of shikonin derivatives to determine their prospects as anticancer agents. They found that **69** (Figure 10) showed remarkable cytotoxic activity against several cell lines, including H1975 (IC_50_ = 1.51 ± 0.42 μM), H1299 (IC_50_ = 5.48 ± 1.63 μM), and HCC827 (IC_50_ = 5.19 ± 1.10 μM).

**Figure 11:**
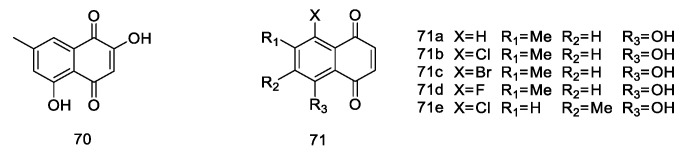
Lapachol derivatives as anticancer agents.

Kishore et al. [11] focused on 7-methyl juglone derivatives from Euclea natalensis, a plant traditionally used in Africa for cancer treatment. These compounds demonstrated significant toxicity against various human cancer cell lines (MCF-7, HeLa, SNO, and DU145), such as a significantly active **70**, with IC_50_ values of 5.3 µM and 6.8 µM (Figure 11), and **71e**, with IC_50_ values of 10.1 µM and 9.3 µM against HeLa and DU145, respectively (Figure 11). The SAR studies reveal that the hydroxyl groups at the 2- and 5-positions play a crucial role in their cytotoxic effects. The study contributes to understanding naturally derived naphthoquinones and their potential in cancer therapy.

**Figure 12:**
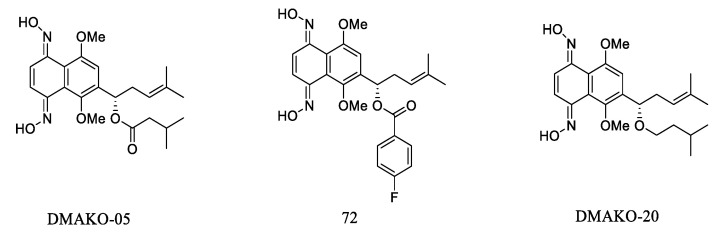
1,4-naphthoquinone oximes as anticancer agents.

In recent studies, we synthesized several naphthoquinone oximes, including both shikonin and alkannin oximes [85,86,87,88]. We assessed their antiproliferative properties against certain cancer cell lines and the normal ones. Most of the prepared naphthoquinone oximes were shown to be strong anticancer agents against the tested cancer cell lines based on the results of biological evaluations. The alkannin oxime (DMAKO-05, Figure 12) with an isovaleryl group as the side chain showed highly potent antiproliferative activity against the human leukemia K562 cells and human breast cancer MCF-7 cells (IC_50_ value of 0.7 μM and 7.5 μM) [86]. Exchange of the aliphatic isobutyl group of DMAKO-05 with an aromatic ring will further potentiate the anticancer activity since **72** (Figure 12) was a five-fold more potent anticancer agent towards MCF-7 cells (IC_50_ = 1.5 μM), as compared with the lead compound. In addition, all of the produced oximes were identified as a group of selective anticancer agents with an IC_50_ value greater than 50 μM for the standard HSF cell line. Cui et al. focused on a new kind of shikonin derivative named DMAKO-20 (Figure 12), which possessed a remarkable antitumor activity against several cancer cell lines, including HCT-15 (IC_50_ = 0.63 ± 0.10 μM), HCT-116 (IC_50_ = 1.08 ± 0.21 μM), and K562 (IC_50_ = 0.4 ± 0.1 μM) [87]. Notably, DMAKO-20 was non-toxic to normal human cells (VEC and HSF). In vivo, experiments indicated that the treatment of DMAKO-20 could prominently diminish the tumor volume. Upon the dosage at 10 mg/kg, the average volume of HCT-15 xenografts (305.8 ± 25.8 mm) was much smaller than that of the control group (59.3% reduction) after 14 days’ treatment. The mechanistic studies demonstrated that the quinone derivative DMAKO-20 underwent tumor-specific CYP1B1-catalyzed oxidation to produce active naphthoquinone mono-oximes and nitric oxide, which exhibited synergistic anticancer effects.

Molecular docking is based on the “lock and key principle” of the interaction between ligands and receptors, simulating the interactions between small molecule ligands and biological macromolecule receptors. The interaction between ligand and receptor is a process of molecular recognition, mainly including electrostatic interaction, hydrophobic interaction, hydrogen-bonding interaction, van der Waals interaction, and so on. The binding mode and affinity between the ligand and corresponding receptor could be predicted through calculation, thereby conducting virtual screening of drugs.

**Figure 13:**
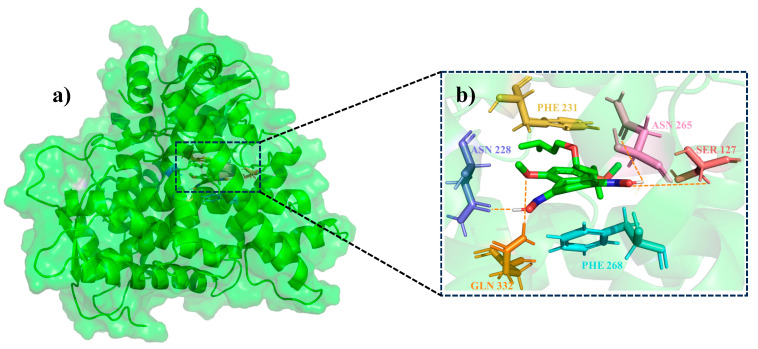
(**a**). The binding mode between **DMAKO-20** and CYP1B1. (**b**). Localized magnification of the binding mode between **DMAKO-20** and CYP1B1. **DMAKO-20** in magenta color was shown in sticks. Ser127 (peach red), Asn228 (violet), Phe231 (ochre), Asn265 (pink), Phe268 (blue), and Gln332 (orange) were shown in sticks. Intercellular hydrogen bonds are represented by orange-yellow dashed lines in the molecular visualization.

To illustrate the binding interaction between quinone oxime DMAKO-20 and CYP1B1, we docked DMAKO-20 into the active site of CYP1B1 (PDB ID: 3PM0). Their binding mode is shown in Figure 13.

Figure 13 demonstrates that **DMAKO-20** binds tightly to the CYP1B1 enzyme, exhibiting a strong binding affinity with a docking score of −9.7 kJ/mol. The oxygen atom at the 1-position of the oxime structure on the **DMAKO-20** naphthalene ring forms a hydrogen bond with Ser127 at a distance of 2.56 Å. Additionally, **DMAKO-20** forms multiple hydrogen bonds with key residues in the CYP1B1 binding site, including Asn265 (1.79 Å) through carbonyl hydrogen bonding, Asn228 (2.04 Å), through oxime hydrogen bonding at position 4, and Gln332 (2.63 Å), through methoxy hydrogen bonding at position 5.

The naphthalene ring of **DMAKO-20** overlaps significantly with the benzene ring directly below Phe231 and above Phe268, forming π-π stacking interactions that enhance binding affinity. The 6-position of the target molecule is relatively small, allowing the flexible hydrophobic side chain to minimize steric hindrance and interact deeply within the CYP1B1 hydrophobic pocket. This hydrophobic interaction further strengthens the binding affinity of **DMAKO-20** to the active site.

**Figure 14:**
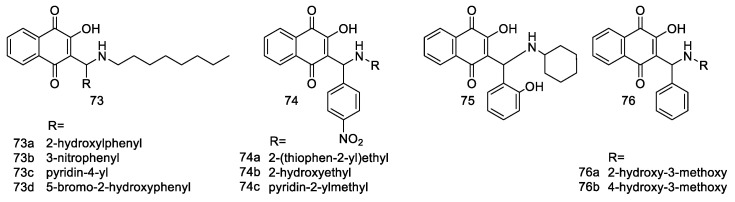
Lawsone derivatives as anticancer agents.

Thakore et al. [89] introduced amino and phenyl groups to the lawsone scaffold in their research. Subsequently, they assessed the anticancer activities of these modified compounds against the HepG2 human liver carcinoma cell line. Among the compounds investigated, precisely **73**–**76** (Figure 14) exhibited significant cytotoxicity against HepG2 cells, with IC_50_ values falling within the range of 1.68 to 11.01 μM. Notably, an analysis of structure-activity relationships revealed that incorporating octyl and hydroxyphenyl groups into the side chain substantially augmented the antitumor activity of these compounds.

Interestingly, it was observed that the introduction of heterocyclic groups at the N-position, as seen in **74a**, **74b**, and **74c,** did not contribute significantly to the cytotoxic activity when compared to the nitrophenyl group in these naphthoquinone compounds. Moreover, the presence of hydrocarbon groups at the N-position and aryl groups at the 1-position of the side chain appeared to enhance the compounds’ antitumor efficacy.

**Figure 15:**
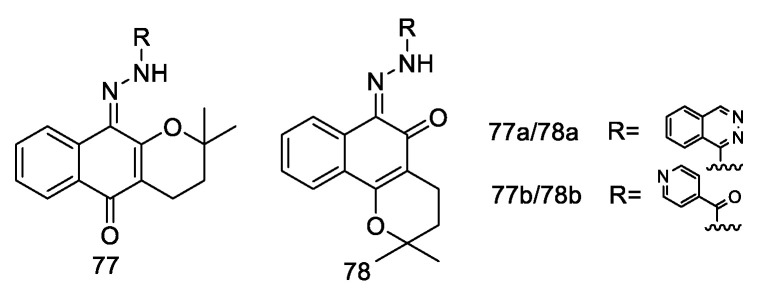
Lapachol derivatives as anticancer agents.

In a related study, Araújo et al. [90] synthesized a series of hydrazone compounds derived from lapachol, with naphthoquinone as the core structure. The researchers then conducted an extensive investigation into the anticancer properties of these synthesized compounds across a panel of human cancer cell lines, including human promyelocytic leukemia (HL-60), human colorectal carcinoma (HCT-16), and human prostate adenocarcinoma (PC-3). Among the compounds evaluated, specifically **77b**, **78a**, and **78b** (Figure 15), demonstrated notable cytotoxic activity against the tested cancer cell lines. Notably, **78a** and **78b** exhibited the highest potency in their anticancer effects.

In addition to assessing their anticancer potential, the researchers also examined the selectivity of these synthetic compounds by testing them on a non-malignant murine fibroblast cell line (L929). The results of this selectivity evaluation highlighted **78a** as the most promising, particularly in its selectivity against HL-60 cells.

The anticancer activity of 1,4-naphthoquinone derivatives is attributed to their ability to induce apoptosis, disrupt redox balance, and inhibit key cellular enzymes. These compounds have shown significant promise across diverse cancer cell lines due to their structural adaptability and selective cytotoxicity.

Juglone derivatives (**48**–**53**) act as potent inhibitors of protein disulfide isomerase (PDI), a critical enzyme involved in protein folding within tumor cells. For instance, **63a** (Figure 8) demonstrated an IC_50_ value of 0.57 µM against RPMI8226 cells, showcasing its potential for treating hematological malignancies [80]. SAR studies reveal that hydroxyl and halogen substitutions enhance ROS generation, amplifying pro-apoptotic activity by inducing oxidative stress, which causes DNA and protein damage, ultimately leading to apoptosis.

Similarly, lawsone derivatives (**73**–**76**) exhibit potent cytotoxic effects against HepG2 (Figure 14 and MCF-7 cell lines. Sulfur-modified lawsone derivatives, in particular, show IC_50_ values ranging from 1.68 to 11.01 µM, demonstrating enhanced activity through improved DNA intercalation and ROS modulation [27,68,89]. These findings confirm that halogen substitution increases cellular uptake and binding affinity to tumor enzymes, improving efficacy, while sulfur modification enhances redox properties, contributing to ROS generation and selective cytotoxicity.

Shikonin and its derivatives exhibit notable anticancer activity through mechanisms such as topoisomerase inhibition [61,62,63,82,83], ROS generation [85], and antiangiogenesis. For example, shikonin oximes (**DMAKO-20**, Figure 12) showed potent activity against HCT-15 and K562 cells, with IC_50_ values as low as 0.4 µM. Molecular docking studies revealed that **DMAKO-20** selectively interacts with CYP1B1, demonstrating strong binding affinity and tumor-specific oxidation, which enhances its anticancer effects [85,86,87,88].

Figure 2, Figure 3, Figure 4, Figure 5, Figure 6, Figure 7, Figure 8, Figure 9, Figure 10, Figure 11, Figure 12, Figure 13, Figure 14 and Figure 15 provide a comprehensive overview of the diverse structures and mechanisms of action of these derivatives. Table 1 complements these data by summarizing IC_50_ values, structural modifications, and mechanisms of action. For instance, halogenated juglone derivatives were particularly effective against drug-resistant cancers, while sulfur-modified vitamin K3 analogs exhibited enhanced [27,68,89] ROS generation and selective cytotoxicity [71].

These findings highlight the versatility and therapeutic potential of 1,4-naphthoquinone derivatives in cancer treatment. Their ability to selectively target cancer cells while minimizing toxicity to normal tissues makes them promising candidates for developing novel anticancer agents targeting drug-resistant malignancies.

### 3.2. Antimicrobial Activity

**Figure 16:**
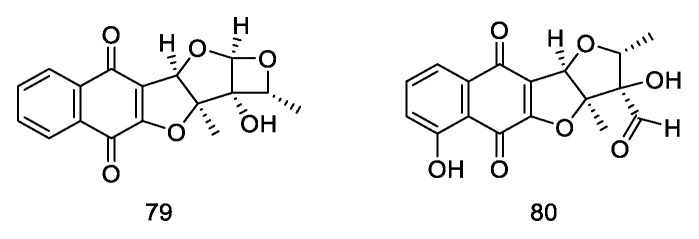
Furo-naphthoquinones as antimicrobial agents.

Khan and Said et al. [91] conducted an isolation study involving a series of furonaphthoquinone compounds **79** and **80** (Figure 16) extracted from the *Amycolatopsis* sp. strain to evaluate their antimicrobial properties. Notably, compounds **79** and **80** demonstrated potent and selective antibacterial activity, particularly against Gram-positive bacteria, compared to the positive controls of Ampicillin and Kanamycin. Compounds **79** and **80** exhibited significantly more inhibition against methicillin-resistant Staphylococcus aureus (MRSA), with MIC values ranging from 4 to 16 μg/mL. However, their antimicrobial activity against the other six tested Gram-positive bacterial strains was comparatively less pronounced. Notably, both compounds **79** and **80** showed limited inhibition against Gram-negative bacteria.

**Figure 17:**
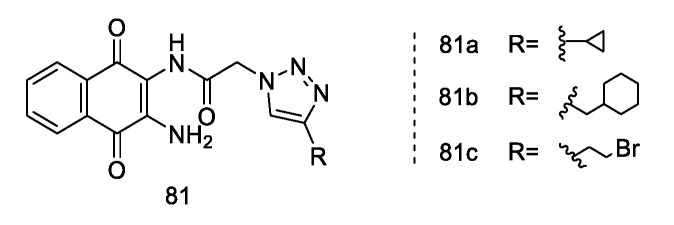
1,4-naphthoquinone derivatives with triazole hybrid groups as antimicrobial agents.

In a separate study, Nural et al. [92] synthesized a series of compounds **81a**–**c** (Figure 17) by introducing triazole hybrid groups at the 2-position of naphthoquinone. Their research aimed to assess the synthesized compound’s antimicrobial activity against different microbial strains and bacterial strains, such as *E. coli*, *B. cereus*, *S. aureus*, *P. aeruginosa*, *E. hirae*, *L. pneumophila* subsp: *pneumophila*, and fungal strains, including *C. albicans* and *C. tropicalis*.

Comparatively, when assessed against the positive control ampicillin, compounds **81a**–**c** exhibited moderate inhibition against the tested bacterial strains. Compounds **81a** and **81b** exhibited potent inhibition against specific microbial strains, with MIC values ranging from 4 to 16 μg/mL. Furthermore, compound **81b** demonstrated moderate inhibition against the evaluated fungal strains compared to the positive antifungal drug.

**Figure 18:**
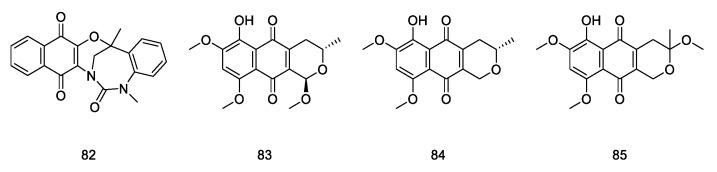
Naphthoquinone derivatives as antimicrobial agents.

Balachandran et al. [93] isolated a novel naphthoquinone derivative bluemomycin **82** (Figure 18) from the Gram-positive filamentous bacterium *Streptomyces* sp. Their research focused on evaluating the antimicrobial activity of bluemomycin against various bacterial strains. Notably, bluemomycin displayed remarkable inhibitory effects, particularly against Gram-negative bacteria. This included strains such as *M. luteus*, *E. aerogenes*, *P. aeruginosa*, *S. typhimurium*, *S. flexneri*, *K. pneumoniae* (ESBL-3894), *K. pneumoniae* (ESBL-75,799), and *E. coli* (ESBL-3984), with MIC values consistently around 12.5 μg/mL. Additionally, bluemomycin exhibited potent antifungal activity against A. flavus, with an MIC value of 6.25 μg/mL.

Supratman et al. [94] investigated naphthoquinone compounds **83**–**85** (Figure 18) obtained from F. napiforme. Their investigation highlighted the substantial inhibitory potential of **83**–**85** against *S. aureus* NBRC 13,276 and *P. aeruginosa* ATCC 15442, with MIC values consistently below 12.5 μg/mL. However, these compounds exhibited limited activity against *A. clavatus* F 318a and *C. albicans* ATCC 2019. Notably, the introduction of hydroxy substitution at the 6-position of naphthoquinone was observed to enhance their antimicrobial activity.

**Figure 19:**
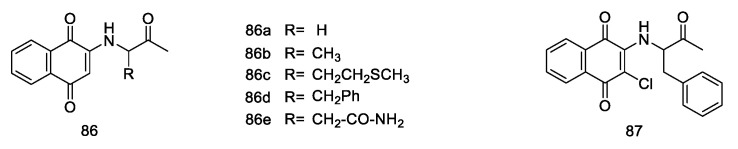
Amino acids modified 1,4-naphthoquinone derivatives as antimicrobial agents.

López-López et al. [95] synthesized naphthoquinone derivatives **86a**–**e** and **87** (Figure 19) enriched with amino acids in their side chains. Their antimicrobial assays revealed that these compounds displayed moderate inhibition against a spectrum of Gram-positive (*E. faecalis* and *S. aureus*) and Gram-negative bacteria (*E. coli* and *P. aeruginosa*), with MIC values consistently below 25 μg/mL. Of particular interest, **83** exhibited potent inhibition against a clinical isolate of *E. coli*, showcasing an MIC value of 24.7 μg/mL. Remarkably, this strain demonstrated substantial resistance to several conventional drugs, including cephalosporins.

**Figure 20:**
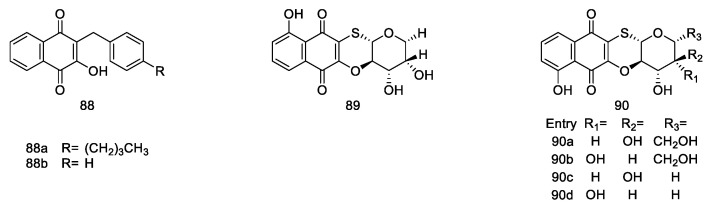
Lawsone derivatives as antimicrobial agents.

In another study, Song et al. [96] engaged in the synthesis of lawsone derivatives **88a** and **88b** (Figure 20) and assessed their efficacy against multidrug-resistant strains, including methicillin-resistant *S. aureus* (MRSA), methicillin-sensitive *S. aureus* (MSSA), and vancomycin-intermediate *S. aureus* (VISA). Their findings indicated that **88a** and **88b** exhibited moderate inhibition against the tested strains. Significantly, 91a demonstrated robust antibacterial activity against MRSA strains, with MIC values ranging from 1.25 to 2.5 μg/mL, a potency comparable to that of positive drugs like vancomycin and daptomycin (MIC = 1 μg/mL) and suppression of other tested strains (MIC value 2.5 μg/mL). Moreover, **88a** was noted for generating ROS and chelating intracellular iron, contributing to its antibacterial activity by disrupting bacterial cell membranes upon entering the cytoplasm.

Sabutski et al. [97] synthesized naphthoquinone–sugar tetracyclic conjugates, with **89** and **90a**–**d** (Figure 20) exhibiting potent inhibition (MIC = 6.25 μM) against the *S. aureus* ATCC 21,027 cell line. Additionally, **90a**–**c** showed moderate antimicrobial activity, highlighting the potential activity enhancement through heterocyclic structures with mercapto–sugars.

**Figure 21:**
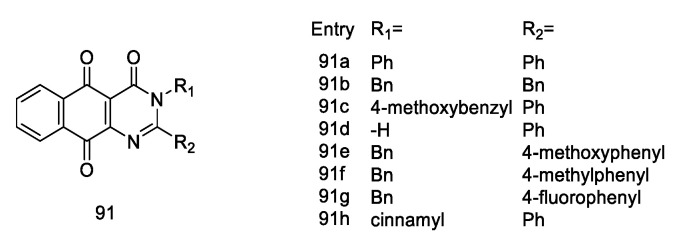
Pyrimidinone-fused naphthoquinone derivatives as antimicrobial agents.

Kim et al. [98] conducted a comprehensive investigation into pyrimidinone-fused naphthoquinone derivatives’ synthesis and antibacterial activity, offering valuable insights into their potential as effective agents against drug-resistant oral bacteria strains. Their meticulous study systematically assessed a range of compounds **91a**–**c** and **91e**–**h** (Figure 21), revealing their impressive inhibitory capabilities across a diverse spectrum of bacterial strains, as evidenced by MIC ranging from 1.56 μg/mL to 12.5 μg/mL. This wide-ranging activity underscores the versatility of these compounds. Notably, the study observed moderate yet substantial antibacterial effects exhibited by **91d** and **91h** against specific bacterial strains. The standout observation was the remarkable inhibition of *P. gingivalis* by **91d**, signifying its potency against this oral bacterial strain. Furthermore, compared to the positive control oxytetracycline, these active compounds consistently demonstrated superior inhibitory properties, extending their effectiveness to critical bacterial strains relevant to oral health, including *E. faecalis*, *S. epidermidis*, *S. mutans* and *P. gingivalis*. The collective findings presented in this study accentuate the promise of pyrimidinone-fused naphthoquinone derivatives as potential therapeutic agents in the battle against drug-resistant oral bacteria strains, offering compelling prospects for future research and clinical applications in oral health management.

**Figure 22:**
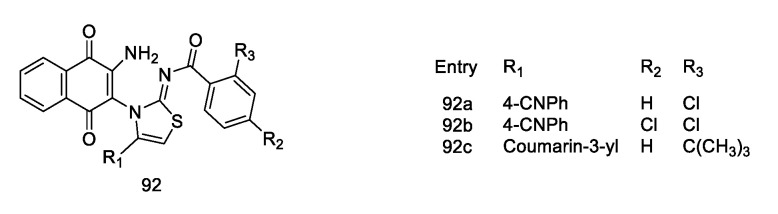
2-iminothiazole substituted naphthoquinone compounds as antimicrobial agents.

Gemili et al. [99] ventured into the synthesis of 2-iminothiazole substituted naphthoquinones **92a**–**c** (Figure 22). These compounds exhibited remarkable antibacterial activity against *M. tuberculosis* H37Rv, with MIC values consistently below 7.81, 3.9 and 1.96 μg/mL, respectively, compared to positive control Isoniazid (MIC value = 0.98 μg/mL) and Ethambutol (MIC value = 1.96 μg/mL).

**Figure 23:**
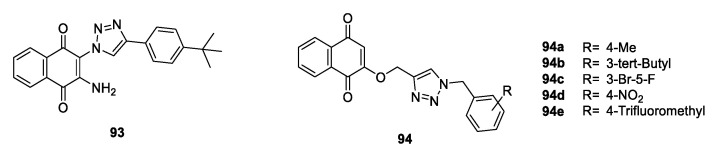
2-amino-3-triazol substituted naphthoquinone compounds as antimicrobial agents.

Erasmus et al. [100] synthesized a series of 2-amino-3-triazol substituted naphthoquinone derivatives **93** and **94a**–**e** (Figure 23). Compounds **93** and **94b** displayed moderate antimycobacterial activity against Mtb, while the rest of the compounds demonstrated potent inhibition, as their MIC values were below or equal to 4.1 μM. Notably, the introduction of electro-positive groups enhances the inhibitory activity against Mtb.

The antimicrobial activity of 1,4-naphthoquinone derivatives is often contingent upon their capacity to accept one or two electrons, forming anionic or double anionic radicals. The presence of electron-donating or electron-withdrawing substituents in 1,4-naphthoquinone determines the production of anionic radicals and the redox properties of the compounds, including the generation of ROS, such as hydrogen peroxide and superoxide, which can damage cells. Consequently, nitrogen and sulfur atoms in the side chain of naphthoquinone play a pivotal role in enhancing the antimicrobial activity of these compounds [101].

Tandon et al. [102] embarked on synthesizing a series of compounds enriched with nitrogen and sulfur atoms, strategically positioned at the 2- or 3-position of the side chain of 1,4-naphthoquinone and incorporated within the ring structure. Their comprehensive investigation thoroughly assessed these compounds’ antimicrobial potential against various pathogens, spanning *C. albicans*, *C. neoformans*, *S. schenckii*, *T. mentagraphytes*, *C. parapsilosis*, and *A. fumigatus*.

**Figure 24:**
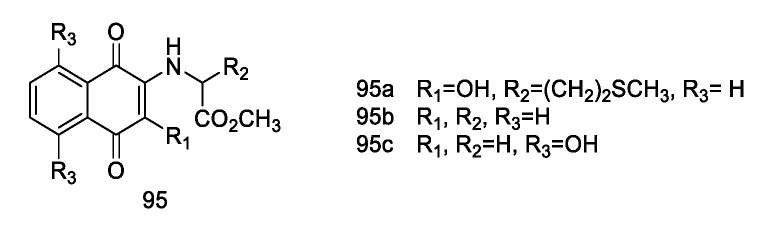
Heteroatoms modified 1,4-naphthoquinone derivatives as antimicrobial agents.

Among the synthesized compounds, **95a** (Figure 24) emerged as a standout, demonstrating remarkable inhibitory activity with a minimum inhibitory concentration (MIC) of 12.5 μg/mL against *C. albicans*. Impressively, this level of inhibition surpassed that of the positive drug Miconazole, which exhibited an MIC of 25 μg/mL against the same pathogen. Furthermore, compound 95a displayed comparable inhibitory efficacy against *C. neoformans* (MIC = 12.5 μg/mL) and *A. fumigatus* (MIC = 12.5 μg/mL) when benchmarked against Miconazole.

The study also unveiled the notable performance of **95b** and **95c**, both exhibiting comparable inhibitory activity against *C. neoformans* (MIC = 12.5 μg/mL) and *C. albicans* (MIC = 25 μg/mL) in comparison to the positive drug Miconazole. Compound **91c** exhibited an intriguingly higher inhibitory potential against *S. schenckii* than the antifungal drug Nystatin, with an MIC value of 13.2 μg/mL.

These compelling findings underscore the promise of these meticulously designed compounds as potent candidates in antimicrobial agents, holding substantial potential for advancing therapeutic approaches against a spectrum of clinically relevant fungal pathogens.

**Figure 25:**
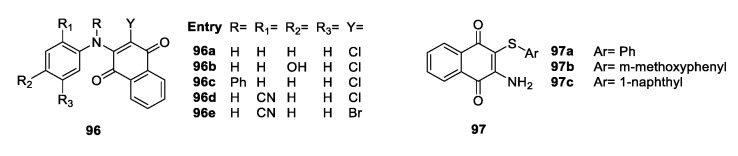
Aromatic group modified 1,4-naphthoquinone derivatives as antimicrobial agents.

Tandon et al. [103] reported that a series of aromatic, sulfur-containing 1,4-naphthoquinone derivatives were synthesized and assessed for their antibacterial and antifungal activities. Among these compounds, **96b**, **97a**, and **97b** (Figure 25) demonstrated robust antifungal effects and notable antibacterial activity against *S. aureus* and *K. pneumoniae*. Intriguingly, compound **96b** exhibited exceptional inhibitory potency against *S. schenckii* (MIC = 1.56 μg/mL), surpassing the performance of clinical antifungal drugs such as Fluconazole, MIC = 2.0 μg/mL, and Miconazole, MIC = 12.5 μg/mL. Furthermore, when compared to the positive drug Amphotericin-B, **96b** consistently exhibited significant inhibitory activity against a spectrum of fungal species, including *C. albicans* MIC = 1.56 μg/mL, *C. neoformans* MIC = 0.78 μg/mL, *T. mentagrophytes* MIC = 1.56 μg/mL, and *A. fumigatus* MIC = 3.12 μg/mL. It is worth noting that compound **96b** is currently undergoing further investigation for toxicological evaluation as a potential lead drug candidate.

Additionally, **97b** displayed comparable inhibitory activity against *C. neoformans* MIC = 12.5 μg/mL, aligning closely with the performance of the positive drug Miconazole. Conversely, compound 100a exhibited superior inhibition against *C. albicans* MIC = 12.5 μg/mL compared to Miconazole MIC = 25 μg/mL, while the activity of compound **100c** closely mirrored that of the positive drug.

The antimicrobial activity of 1,4-naphthoquinone derivatives is primarily attributed to their ability to generate ROS and disrupt key biological processes, including enzyme inhibition and biofilm disruption [39,40]. These compounds demonstrate broad-spectrum efficacy against drug-resistant bacterial and fungal pathogens. Their mechanisms of action involve ROS generation, which induces oxidative stress, causing DNA, protein, and membrane damage [74,96,98]. Structural modifications such as electron-donating and electron-withdrawing substituents significantly enhance their potency by modulating electron transfer properties, while sulfur and nitrogen substitutions improve redox potential and enable selective microbial targeting.

Studies have highlighted the structural diversity and biological activity of 1,4-naphthoquinone derivatives. For instance, **79** and **80**, isolated from *Amycolatopsis* sp., exhibit selective antibacterial activity against Gram-positive bacteria, including methicillin-resistant *Staphylococcus aureus* (MRSA), with MIC values ranging from 4 to 16 µg/mL (Figure 16) [91]. Similarly, triazole-hybrid derivatives **81a**–**c** (Figure 17) demonstrated moderate inhibition against bacterial strains such as *E. coli* and *S. aureus*, and fungal strains including *Candida albicans*, with MIC values between 4 and 16 µg/mL [92]. Notably, the sulfur-modified naphthoquinone bluemomycin (**82**, Figure 24), isolated from *Streptomyces* sp., exhibited potent activity against Gram-negative bacteria and fungi, including *E. coli* and *Aspergillus flavus*, with MIC values as low as 6.25 µg/mL [93].

In another study, lawsone derivatives **88a** and **88b** (Figure 20) displayed robust activity against multidrug-resistant strains, including MRSA, MSSA, and VISA [96]. Compounds **88a** and **91a** exhibited MIC values between 1.25 and 2.5 µg/mL, comparable to clinically used drugs, such as vancomycin and daptomycin [96,98]. These derivatives demonstrated a dual mechanism of action involving ROS generation and intracellular iron chelation, which disrupts bacterial membranes. Additionally, pyrimidinone-fused naphthoquinones (Figure 21) effectively inhibited oral pathogens, such as *P. gingivalis* and *S. mutans*, with MIC values ranging from 1.56 to 12.5 µg/mL, showcasing their potential for oral health applications [97].

Compounds enriched with sulfur and nitrogen substitutions have demonstrated significant antifungal activity. For example, compound **95a** (Figure 24) exhibited superior inhibitory activity against *C. albicans*, *C. neoformans*, and *Aspergillus fumigatus*, with MIC values consistently around 12.5 µg/mL. Compared to the clinical antifungal drug miconazole, **95a** demonstrated higher efficacy against *C. albicans*. Another notable compound, **96b** (Figure 25), showed exceptional potency against *S. schenckii* (MIC = 1.56 µg/mL) and other fungal pathogens, outperforming conventional drugs such as fluconazole and amphotericin-B [102].

Figure 16, Figure 17, Figure 18, Figure 19, Figure 20, Figure 21, Figure 22, Figure 23, Figure 24 and Figure 25 illustrate the structural diversity and mechanisms of action of these derivatives, emphasizing the role of substituents in determining their efficacy. Table 2 complements these findings by summarizing the MIC values, structural modifications, and mechanisms of action for key compounds. For instance, halogenated juglone derivatives exhibited remarkable activity against drug-resistant bacterial strains, while sulfur-modified lawsone derivatives excelled in antifungal applications.

These findings underscore the versatility of 1,4-naphthoquinone derivatives as potent antimicrobial agents. Their structural tunability, combined with their ability to selectively target microbial cells, positions them as promising candidates for the development of novel therapies to combat multidrug-resistant pathogens.

### 3.3. Antiviral Activity

**Figure 26:**
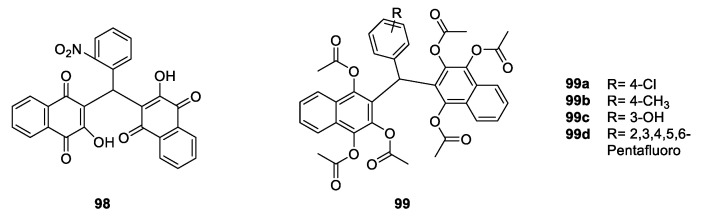
Bis-naphthoquinone compounds as antiviral agents.

In the realm of medicinal chemistry, Gonzaga, Daniel T. G., et al. [104] made significant strides in antiviral research by synthesizing a series of bis-naphthoquinone compounds, focusing on their activity against the Zika virus. They identified several high-activity **98** and **99a**–**d** (Figure 26). These compounds were found to exhibit potent antiviral properties, with EI_50_ values at or below 1.38 µM, notably surpassing the efficacy of the standard antiviral agent ribavirin, which has an EI_50_ value of 2.98 µM.

**Figure 27:**
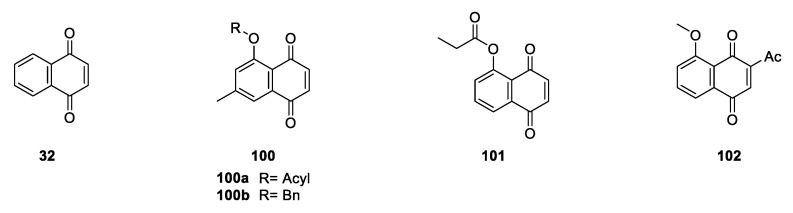
Juglone derivatives as antiviral agents.

In a related study, our lab [105] explored the antiviral potential of juglone derivatives against SARS-CoV-2. We discovered that **32** and **100**–**102** (Figure 27) showed remarkable inhibitory effects on SARS-CoV-2 Mpro. This enzyme is vital for the virus’s replication and transcription within host cells. The tested compounds demonstrated IC_50_ values ranging from 72.07 to 220.9 nM. Compared to the positive control shikonin, with an IC_50_ value of 15.75 ± 8.22 µM, **100**–**102** exhibited more robust activity against SARS-CoV-2 M^pro^. Compound 106 notably emerged as the most potent inhibitor, with an IC_50_ value of 72.07 nM.

Additionally, compound **101** significantly suppressed the proliferation of SARS-CoV-2 M^pro^ in vitro (EC_50_ = 4.55 µM). The molecular docking assays revealed that these compounds interact effectively with the enzyme’s active site, primarily through hydrogen bonding with adjacent amino residues. Our study also provided insights into structure-activity relationships, suggesting that the incorporation of an acyl group or benzyl group enhances antiviral efficacy. In contrast, adding a methyl group, methoxy group, or the annulation of the naphthoquinone core with a phenyl group decreases the inhibition activities.

**Figure 28:**
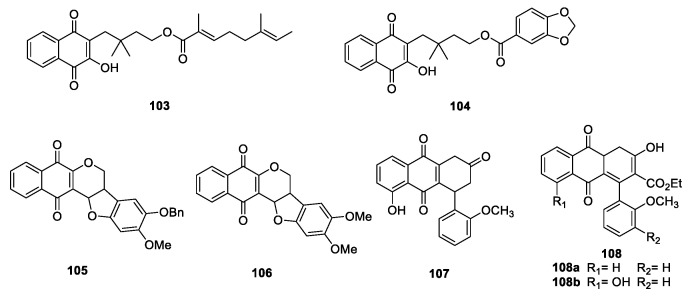
1,4-naphthoquinone derivatives and lapachol derivatives as antiviral agents.

Naphthoquinone derivatives are the subject of considerable interest to the scientific community throughout the globe, particularly concerning their impact on virus replication and immune response modulation [106]. For instance, Sendl et al. [107] isolated two novel 1,4-naphthoquinone-active compounds, Rhinacanthin-C **103** (Figure 28) and Rhinacanthin-D **104** from *Rhinacanthus nasutus*. These compounds exhibited significant antiviral activity against cytomegalovirus (CMV), with EC_50_ values of 0.02 μg/mL and 0.22 μg/mL, respectively. Alcides et al. [108] synthesized derivatives **105** and **106** using lapachol (tobacco genus) as a lead compound and found them to be significantly effective against Herpes Simplex Virus-2, with IC_50_ values of 180 ng/mL and 25 ng/mL, respectively. This highlights the potential for developing a broader antiviral spectrum through chemical modifications and animal models of viral infection studies.

Additionally, HIV-1 reverse transcriptase inhibitors are of remarkable interest to medicinal chemists, such as the aryl-substituted naphthoquinone compounds synthesized by Ilina et al. [109], which showed potent inhibition against four reverse transcriptase M(215), M(67), and M(67, 70, 215) in HIV-1 recombinant wild-type and mutant forms. Compounds **107**, **108a**, and **108b** (Figure 28 were particularly effective, indicating that 1,4-naphthoquinone derivatives are potent inhibitors of HIV-1 reverse transcriptase. Furthermore, some compounds inhibit azidothymidine-resistant wild-type and mutant reverse transcriptase. This indicates that 1,4-naphthoquinone derivatives may offer an alternative mechanism of action to existing drugs like nevirapine.

**Figure 29:**
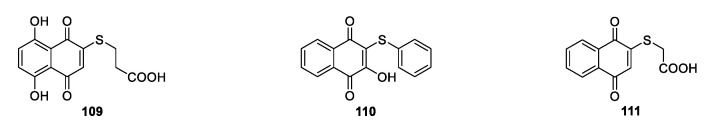
Sulfur-modified 1,4-naphthoquinone derivatives as antiviral agents.

A, V.K.T. et al. [110] synthesized 2-substituted-1,4-naphthoquinone derivatives with sulfur atoms in their side chains, demonstrating significant inhibitory activity against influenza-A and HSV-1 viruses with **109** and **110** (Figure 29). Simultaneously, they also synthesized **111** from 1,4-naphthoquinone, showing notable activity against type II poliovirus.

**Figure 30:**
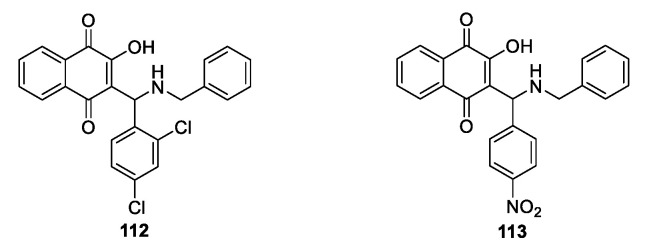
2-aminomethyl-3-hydroxy-1,4-naphthoquinones derivatives as antiviral agents.

Behl et al. synthesized a series of 2-aminomethyl-3-hydroxy-1,4-naphthoquinones derivatives. Subsequently, they encapsulated these quinones in liposomes for antiviral testing against HSV-1. The study revealed that these aminomethyl naphthoquinones derivatives, when enclosed within phosphatidylcholine liposomes, effectively inhibited HSV-1 replication. Notably, derivatives featuring a benzyl group (**112,** Figure 30) and a nitrobenzene moiety (**113**) demonstrated selective index values over nine times greater than those of acyclovir, used as the positive control. The EC_50_ values for **112** and **113** were 0.56 ± 0.02 µM and 0.36 ± 0.04 µM, respectively, indicating a potency almost four and nine times higher than acyclovir, which showed an EC_50_ of 3.16 ± 0.09 µM under identical experimental conditions.

**Figure 31:**
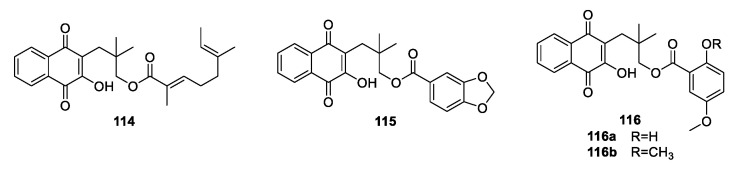
Rhinacanthins as antiviral agents.

Ngoc et al. conducted a study to evaluate the antiviral efficacy of rhinacanthins C (**114**), D (**115**), N (**116**) (Figure 31), extracted from the roots of Rhinacanthus Nasutus—a medicinal plant of the *Acanthaceae* family traditionally used in treating herpes virus infections [111]. The in vitro experiments demonstrated that all four compounds inhibited the influenza virus A/PR/8/34 (H1N1). The mean IC_50_ values were recorded as 0.30 µM for rhinacanthin C (**114**), 0.95 µM for rhinacanthin D (**115**), and 1.95 µM for rhinacanthin N (**116**).

**Figure 32:**
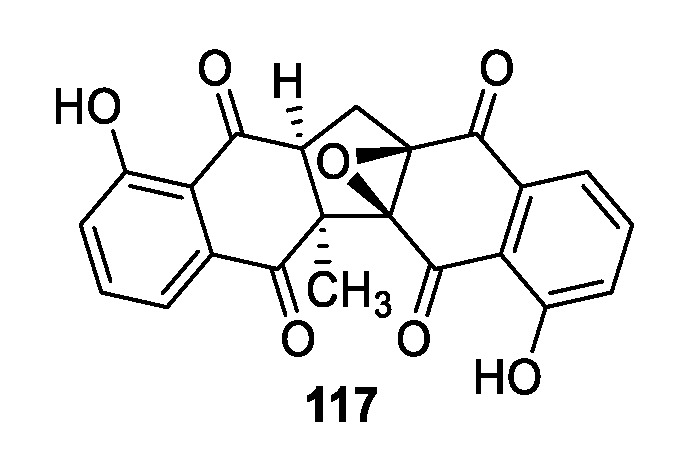
Novel natural 1,4-naphthoquinones products as antiviral agents.

Furthermore, Lisseth Cetina-Montejo and colleagues successfully isolated several novel naphthoquinones from the stem bark of *Diospyros anisandra*, with a specific focus on zeylanone epoxide (ZEP) (**117**, Figure 32) for antiviral activity assessment [112]. The team conducted experiments by infecting MDCK cells with strains H1N1, H1N1-H275Y, and H3N2, followed by treatment with ZEP. Virus titers were quantified using a plaque assay. The research revealed mean IC_50_ values of 0.65 µM for H1N1, 2.77 µM for H1N1-H275Y, and 1.6 µM for H3N2.

**Figure 33:**
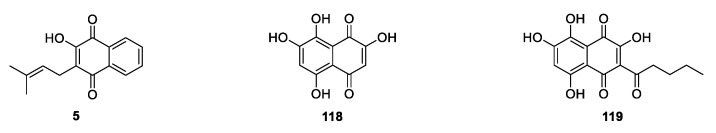
Natural naphthoquinone compounds as antiviral agents.

Zima and colleagues focused on the isolation of natural naphthoquinones, specifically lapachol (**5**), mompain (**118**), and quambalarine (**119**) (Figure 33) from the *Quambalaria cyanescens* fungus [113]. Their investigation into the antiviral properties of these compounds against H1N1 revealed varying levels of efficacy. Lapachol exhibited moderate inhibitory activity with an IC_50_ value of 19 μM. In contrast, mompain and quambalarine demonstrated significant potency as sub-micromolar inhibitors, with IC_50_ values of 0.43 μM and 0.29 μM, respectively.

**Figure 34:**
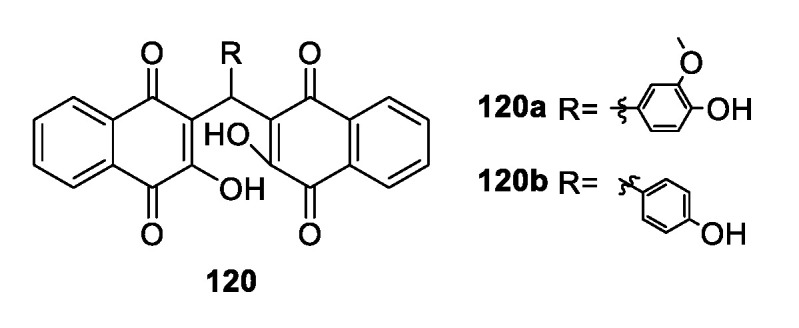
1,4-Naphthaquinone dimers as antiviral agents.

Garima and colleagues embarked on a project to synthesize a series of compounds, subsequently testing their efficacy against the neuraminidase (NA) of the H5N1 virus [114]. Among these synthesized compounds, **120a** (Figure 34) and compound **120b** emerged as the most potent in terms of antiviral activity. Specifically, compound 123a exhibited an IC_50_ of 29 ± 0.9 μM, while **120b** demonstrated an IC_50_ of 26.5 ± 0.7 μM.

**Figure 35:**
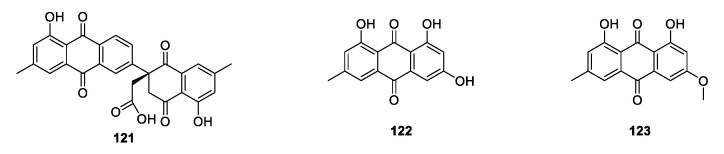
Natural anthraquinone analogs as antiviral agents.

Xu et al. successfully isolated three anthraquinone analogs from the roots of *P. odoratum* [115]. Utilizing NMR, MS, and CD techniques, they identified several novel natural naphthoquinones, notably compound **121** (Figure 35). Additionally, compounds emodin (**122**) and physcion (**123**, Figure 35) were also detected in the extracts. The team then proceeded to evaluate the antiviral efficacy of these compounds against the influenza A virus (H1N1). Their findings indicated IC_50_ values of 11.4 μM for compound **121**, 11.0 μM for emodin (**122**), and notably lower at 2.3 μM for physcion (**123**).

The antiviral activity of 1,4-naphthoquinone derivatives is rooted in their ability to inhibit viral enzymes, disrupt replication pathways, and modulate immune responses. These compounds have shown significant potential against a wide range of viruses, including SARS-CoV-2 [105], Zika virus [104], cytomegalovirus (CMV) [107], herpes simplex virus (HSV), and influenza strains [110].

Bis-naphthoquinone derivatives (**98** and **99**) have demonstrated exceptional efficacy against the Zika virus, achieving EI_50_ values at or below 1.38 µM, significantly surpassing the activity of ribavirin (EI_50_ = 2.98 µM) (Figure 26) [104]. Their dimeric structure enhances interactions with viral replication proteins, contributing to their potent antiviral effects. Similarly, juglone derivatives, such as **100**–**103** (Figure 27), exhibit strong inhibition of SARS-CoV-2 main protease (Mpro), a key enzyme for viral replication [106]. Compound **100** demonstrated an IC_50_ of 72.07 nM, far exceeding the activity of shikonin (IC_50_ = 15.75 ± 8.22 µM). Molecular docking studies reveal that these compounds form hydrogen bonds at the enzyme’s active site, with structural modifications like acyl and benzyl groups enhancing efficacy [105].

1,4-Naphthoquinone derivatives also show notable activity against DNA and RNA viruses. Rhinacanthin-C and Rhinacanthin-D (Figure 28), isolated from *Rhinacanthus nasutus*, demonstrated potent effects against CMV and HSV, with EC_50_ values as low as 0.02 µg/mL and 0.22 µg/mL, respectively [107]. Similarly, lapachol derivatives, such as mompain and quambalarine (Figure 31, exhibited sub-micromolar inhibition of influenza A (H1N1), with IC_50_ values of 0.43 µM and 0.29 µM, respectively [113]. These findings underscore the importance of structural features, such as sulfur and nitrogen substitutions, which enhance redox activity and membrane penetration.

Further studies have highlighted the versatility of these derivatives against resistant viral strains. Sulfur-modified derivatives, including **109** and **110** (Figure 29), were highly effective against HSV-1 and influenza A, emphasizing the role of sulfur in boosting antiviral efficacy [110]. Additionally, rhinacanthins extracted from *Rhinacanthus nasutus* showed strong inhibition of influenza A (H1N1), with IC_50_ values ranging from 0.30 to 1.95 µM (Figure 31) [111]. Structural features such as acyl and benzyl substitutions improved binding affinity, while bulky or electron-donating groups like phenyl or methoxy reduced activity, as shown through SAR analysis.

Figure 26, Figure 27, Figure 28, Figure 29, Figure 30, Figure 31, Figure 32, Figure 33, Figure 34 and Figure 35 illustrates the structural diversity and mechanisms of action of 1,4-naphthoquinone derivatives, while Table 3 summarizes their key activities, IC_50_ values, and structural modifications. For instance, juglone derivatives targeting SARS-CoV-2 M^pro^ exhibited IC_50_ values as low as 72.07 nM, while rhinacanthins and lapachol derivatives showed exceptional potency against influenza A. These findings highlight the potential of 1,4-naphthoquinone derivatives as versatile antiviral agents. Their structural tunability and ability to selectively target viral systems make them promising candidates for combating emerging viral pathogens.

### 3.4. Miscellaneous

**Figure 36:**
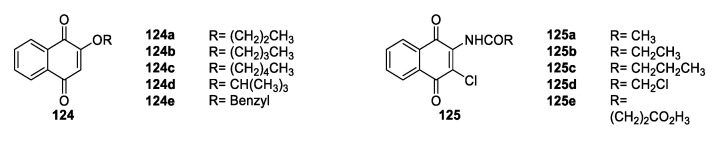
The chemical structure of 1,4-naphthoquinone derivatives.

Lien et al. [116] synthesized a series of 2-substituted aliphatic and aromatic 1,4-naphthoquinone derivatives. These derivatives effectively inhibited the formation of neutrophil superoxide anion, demonstrating substantial anti-inflammatory activity. Notably, **124a** and **124b** (Figure 36) achieved IC_50_ values of 0.6 μM, exhibiting inhibition intensity 25 times greater than the positive control, trifluoperazine. Additionally, these compounds showed promising antiplatelet agglutination activity. Mass cells release various inflammatory regulatory factors during immune responses, crucial in the passive skin allergic response (PCA). In a related study, Huang et al. [117] synthesized amide-substituted 1,4-naphthoquinone derivatives, assessing their antiallergic activity. **125a**–**125c** significantly inhibited mast cell growth and degranulation at 0.3–1 μg/mL concentrations, thus showing notable antiallergic potential. Other biological activities of 1,4-naphthoquinones, such as antithrombotic, free radical scavenging, and antiradical activities, have been reported [45].

## 4. Conclusions and Further Perspectives

The exploration and structural modification of lead compounds derived from natural products is a cornerstone of new drug development. Among these, natural 1,4-naphthoquinone derivatives have garnered significant attention for their diverse biological activities, including antitumor, antibacterial, antiviral, and anti-inflammatory effects. However, the extensive cytotoxicity of 1,4-naphthoquinones has limited their clinical applications, primarily due to non-specific damage to both cancerous and normal cells.

From a chemical perspective, the anticancer activity and pervasive toxicity of naphthoquinones are closely linked to their ability to ROS and induce bio-reductive alkylation. These processes cause non-specific damage to biological macromolecules, such as nucleic acids and proteins, compromising the selectivity of these compounds. Innovations in structural modification have sought to address these limitations, with particular success seen in shikonin oximes. Derived from the bioactive component of the traditional Chinese medicine *Lithospermum erythrorhizon*, shikonin oximes function as tumor-specific CYP1-activated prodrugs, significantly enhancing selectivity and reducing cytotoxicity. This prodrug strategy has demonstrated potent anticancer activity both in vivo and in vitro, offering a promising pathway for mitigating the inherent limitations of 1,4-naphthoquinones. Additionally, naphthoquinone derivatives, including alkannin, lawsone, and plumbagin derivatives exhibited remarkable antimicrobial activity, derived from their generation of ROS. Naphthoquinone derivatives also possess antiviral activity both against DNA and RNA viruses, based on numerous mechanisms, including topoisomerase inhibition and DNA intercalation.

Rational structural modifications of the quinone scaffold have focused on introducing substituents, such as halogen, methyl, methoxy, and hydroxyl groups, at strategic positions on the quinone ring or altering the ring itself. These modifications have yielded compounds with enhanced activity; however, they can also exacerbate toxicity. The development of selective, tumor-targeted quinones remains a critical goal, balancing the dual objectives of improving antitumor efficacy and reducing cytotoxicity.

These advances in structural modification highlight the potential of 1,4-naphthoquinones as scaffolds for next-generation anticancer drugs, emphasizing the need for continued innovation to unlock their clinical potential.

## Figures and Tables

**Table 1:** Anticancer mechanisms of 1,4-naphthoquinone derivatives.

Compound	Mechanism of Action	Notable Findings	Ref.
Juglone (**2**)	ROS generation, DNA fragmentation	Effective against melanoma and colorectal cancer; disrupts bacterial biofilms.	[39,40,80]
Plumbagin (**5**)	Mitochondrial ROS generation, apoptosis	Potent activity against pancreatic and breast cancer cells; antifungal activity.	[74]
Shikonin (**6**)	Topoisomerase inhibition, antiangiogenesis	Inhibits tumor growth; effective against resistant cancer cells and viral pathogens.	[13,65,84]
β-Lapachone	NAD(P)H:quinone oxidoreductase activation	Promotes apoptosis in tumor cells; selective for cancer cells over normal tissues.	[45]
Alkannin (**7**)	Topoisomerase inhibition, anti-inflammatory	Strong antiproliferative activity; selective toxicity towards cancer cells.	[56,60,61]
Lapachol (**4**)	DNA intercalation, ROS-mediated DNA damage	Effective against liver and cervical cancers; potent activity against HSV-2 virus.	[90]
Vitamin K2 (**1**)	Ferroptosis inhibition, apoptosis induction	Synergizes with other anticancer agents; regulates calcium metabolism.	[71]
Sulfur-Modified Vitamin K3 Analogs (**51**)	Enhanced ROS generation, apoptosis induction	Highly selective for cancer cells over normal human cells (e.g., WI-38).	[72]
Phenylaminophenyl-thiosulfone Compounds (**49**)	Cytotoxicity via DNA damage	Effective against HeLa cells; IC_50_ comparable to doxorubicin.	[71]
Juglone Derivatives (PDI Inhibitors) (**71**)	Protein disulfide isomerase inhibition	Effective against MDA-MB-231 (IC_50_ = 6.96 µM) and RPMI8226 (IC_50_ = 0.57 µM).	[72]
Shikonin Oximes (**DMAKO-20**)	Tumor-specific CYP1B1-catalyzed oxidation	Highly effective against leukemia and breast cancer cells (IC_50_ = 0.70 µM).	[85,86,87,88]
Lawsone Derivatives (**73**–**76**)	Hydroxylation and DNA intercalation	Potent against HepG2 cells; IC_50_ values range from 1.68 to 11.01 µM.	[28,68,89]
Lapachol Hydrazones (**77**, **78**)	Cell cycle arrest, DNA synthesis inhibition	Selective for HL-60 leukemia cells.	[90]

**Table 2:** Antimicrobial activity of juglone and lawsone derivatives against bacterial and fungal pathogens.

Compound	Mechanism of Action	Notable Findings	Ref.
Lawsone (**4**)	Topoisomerase I inhibition, UV protection	Potential as a photoprotective agent; inhibits DNA replication in pathogens.	[28,49]
Plumbagin (**4**)	Mitochondrial ROS generation, apoptosis	Potent activity against pancreatic and breast cancer cells; antifungal activity.	[74]
Alkannin (**7**)	Topoisomerase inhibition, anti-inflammatory	Strong antiproliferative activity; selective toxicity towards cancer cells.	[56,60,61]
Naphthoquinone-Sugar Conjugates (**89**, **90**)	Cell wall disruption	Potent against methicillin-resistant *S. aureus* (MIC = 6.25 μM).	[97]
Furo-Naphthoquinones (**91**)	ROS generation and DNA disruption	Effective against *S. aureus* and MRSA (MIC as low as 4 µg/mL).	[98]
Bluemomycin (**82**)	ROS-mediated bacterial inhibition	Active against Gram-negative bacteria (e.g., *E. coli*, *P. aeruginosa*).	[93]
Pyrimidinone-Fused Naphthoquinones (**91**)	Disrupts bacterial membranes	Effective against *P. gingivalis* (MIC = 1.56 µg/mL); potential for oral health applications.	[98]

**Table 3:** Antiviral activities of 1,4-naphthoquinone derivatives, including IC_50_ values and structural modifications.

Compound	Mechanism of Action	Notable Findings	Ref.
Shikonin (**6**)	Topoisomerase inhibition, antiangiogenesis	Inhibits tumor growth; effective against resistant cancer cells and viral pathogens.	[13,65,84]
Vitamin K2 (**1**)	Ferroptosis inhibition, apoptosis induction	Synergizes with other anticancer agents; regulates calcium metabolism.	[69,71]
Rhinacanthin C (**104**)	Viral DNA replication inhibition	Active against cytomegalovirus and influenza strains.	[107]
Rhinacanthin D (**105**)	DNA intercalation, replication inhibition	Potent activity against herpes simplex virus and influenza.	[111]
Mompain (**118**)	ROS-mediated viral inhibition	Highly effective against H1N1 virus (IC_50_ = 0.43 μM).	[113]
Quambalarine (**119**)	DNA intercalation	Potent against influenza A virus (IC_50_ = 0.29 μM).	[113]
Juglone Derivatives (**100**–**103**)	SARS-CoV-2 Mpro inhibition	IC_50_ values as low as 72.07 nM; effective against SARS-CoV-2 replication.	[105]
Bis-Naphthoquinones (**98**, **99**)	Viral replication inhibition	Potent against Zika virus (EI_50_ ≤ 1.38 µM).	[104]
